# Structural, Spectroscopic, Electric and Magnetic Properties of New Trigonal K_5_FeHf(MoO_4_)_6_ Orthomolybdate

**DOI:** 10.3390/molecules28041629

**Published:** 2023-02-08

**Authors:** Victoria Grossman, Victor Atuchin, Bair G. Bazarov, Aleksandr Aleksandrovsky, Evgeniy Eremin, Alexander Krylov, Natalia Kuratieva, Jibzema G. Bazarova, Nikolai Maximov, Maxim Molokeev, Aleksandr Oreshonkov, Natalia Pervukhina, Nikolay Shestakov

**Affiliations:** 1 Laboratory of Oxide Systems, Baikal Institute of Nature Management, SB RAS, Ulan-Ude 670047, Russia; 2 Laboratory of Optical Materials and Structures, Institute of Semiconductor Physics, SB RAS, Novosibirsk 630090, Russia; 3 Department of Applied Physics, Novosibirsk State University, Novosibirsk 630090, Russia; 4 Research and Development Department, Kemerovo State University, Kemerovo 650000, Russia; 5 Department of Industrial Machinery Design, Novosibirsk State Technical University, Novosibirsk 630073, Russia; 6 R&D Center “Advanced Electronic Technologies”, Tomsk State University, Tomsk 634034, Russia; 7 Laboratory of Coherent Optics, Kirensky Institute of Physics, Federal Research Center KSC SB RAS, Krasnoyarsk 660036, Russia; 8 Institute of Nanotechnology, Spectroscopy and Quantum Chemistry, Siberian Federal University, Krasnoyarsk 660041, Russia; 9 Kirensky Institute of Physics, Federal Research Center KSC SB RAS, Krasnoyarsk 660036, Russia; 10 School of Engineering Physics and Radio Electronics, Siberian Federal University, Krasnoyarsk 660041, Russia; 11 Laboratory of Molecular Spectroscopy, Kirensky Institute of Physics, Federal Research Center KSC SB RAS, Krasnoyarsk 660036, Russia; 12 Laboratory of Crystal Chemistry, Institute of Inorganic Chemistry, SB RAS, Novosibirsk 630090, Russia; 13 Institute of Chemistry and Chemical Technology, Federal Research Center KSC SB RAS, Krasnoyarsk 660036, Russia; 14 Department of Physics, Far Eastern State Transport University, Khabarovsk 680021, Russia; 15 School of Engineering and Construction, Siberian Federal University, Krasnoyarsk 660041, Russia

**Keywords:** ternary molybdate, phase relations, crystal structure, Raman, electronic structure, magnetic properties

## Abstract

A new multicationic structurally disordered K_5_FeHf(MoO_4_)_6_ crystal belonging to the molybdate family is synthesized by the two-stage solid state reaction method. The characterization of the electronic and vibrational properties of the K_5_FeHf(MoO_4_)_6_ was performed using density functional theory calculations, group theory, Raman and infrared spectroscopy. The vibrational spectra are dominated by vibrations of the MoO_4_ tetrahedra, while the lattice modes are observed in a low-wavenumber part of the spectra. The experimental gap in the phonon spectra between 450 and 700 cm^−1^ is in a good agreement with the simulated phonon density of the states. K_5_FeHf(MoO_4_)_6_ is a paramagnetic down to 4.2 K. The negative Curie–Weiss temperature of −6.7 K indicates dominant antiferromagnetic interactions in the compound. The direct and indirect optical bandgaps of K_5_FeHf(MoO_4_)_6_ are 2.97 and 3.21 eV, respectively. The K_5_FeHf(MoO_4_)_6_ bandgap narrowing, with respect to the variety of known molybdates and the ab initio calculations, is explained by the presence of Mott-Hubbard optical excitation in the system of Fe^3+^ ions.

## 1. Introduction

The design of complex molybdate crystals containing different cations attracts a lot of attention because it provides an opportunity to develop a new functionality of material properties. Over the past few decades, many molybdate compounds with various combinations of cations have been studied to see their structural and chemical properties and evaluate the potential for practical applications [1,2,3,4,5,6,7,8,9,10,11]. Among the complex molybdates, the compounds containing transition metals are of significant interest because valuable optical, electrical, magnetic and catalytic characteristics can be reached in such materials [1,12,13,14,15,16,17,18,19,20,21,22]. In this relation, the rich crystal family with the general composition *A*_5_*MT*(MoO_4_)_6_ (*A* = K, Tl, Rb, Cs; *M* = In, Sc, Bi, *Ln*; *T* = Zr, Hf) is promising for searching for new efficient materials [21] because the trigonal structure contains two mixed positions for the (*M*,*T*) ions, which provides a flexibility for different cation accommodations. At present, the crystal structures are known for several compounds from this crystal family [21,23,24,25,26,27,28,29,30,31] and their field in the space of the unit cell parameters have been determined [21]. Furthermore, the formation of several other compounds of the same structural type has been detected, but their structural parameters remain unknown [32,33,34,35].

In trigonal *A*_5_*MT*(MoO_4_)_6_ compounds, the lowest cell parameter values were obtained in K_5_InHf(MoO_4_)_6_ [23], and, from the structural point of view, it is very interesting to determine the low boundary for the stability of this trigonal structure. From our test experiments, it is found that the structure in the space group *R*$\bar{3}$*c* is not formed for *A* = Li, Na and *T* = Ti, and these cations are not appropriate for use in *A*_5_*MT*(MoO_4_)_6_ compounds. Thus, the only possibility to decrease the cell parameters in *A*_5_*M*(Zr,Hf)(MoO_4_)_6_ is to combine K^+^ and *M*^3+^ ions, where the effective ion radius of the *M*^3+^ ion is lower than that of the In^3+^ ion. Accordingly, in the present work, the ternary system K_2_MoO_4_–Fe_2_(MoO_4_)_3_–Hf(MoO_4_)_2_ is considered in the search for the formation of trigonal K_5_FeHf(MoO_4_)_6_. As it is known, the Fe^3+^ ion radius is significantly lower than that of the In^3+^ ion and the substitution of Fe^3+^ for In^3+^ in K_5_InHf(MoO_4_)_6_ may induce a new trigonal crystal with lower cell parameters [36]. Commonly, oxide crystals containing Fe^3+^ ions are characterized by interesting structural, electronic, magnetic and catalytic properties [16,17,18,22,37,38,39,40,41,42,43], and such effects can be assumed in the designed K_5_FeHf(MoO_4_)_6_ crystal.

## 2. Results and Discussions

### 2.1. Subsolidus Phase Relations in the K_2_MoO_4_–Fe_2_(MoO_4_)_3_–Hf(MoO_4_)_2_ System

The boundary K_2_MoO_4_–Fe_2_(MoO_4_)_3_ system was characterized by the formation of double molybdates KFe(MoO_4_)_2_, K_3_Fe(MoO_4_)_3_ and K_5_Fe(MoO_4_)_4_ [44,45,46,47]. In the structures, the molybdenum atoms are tetrahedrally coordinated, while the Fe^3+^ cations usually have the octahedral coordination. Double molybdate K_5_Fe(MoO_4_)_4_ was found to be dimorphic [48]. The high-temperature modification crystallizes in the layer structure of palmierite K_2_Pb(SO_4_)_2_ [49]. KFe(MoO_4_)_2_ belongs to the family of layered trigonal molybdates [50,51,52,53,54]. KFe(MoO_4_)_2_ has gained considerable attention, as this compound constitutes an example of the nearly two-dimensional ‘triangular’ antiferromagnet available in a single crystal form [55]. KFe(MoO_4_)_2_ also exhibits ferroelastic phase transitions [56]. The crystal structure of KFe(MoO_4_)_2_ at ambient pressure and above 312 K is related to space group *P*$\bar{3}$*m*1. Below 312 K, the crystal structure is monoclinic. Upon increasing the pressure, the monoclinic phase transforms to the *P*$\bar{3}$*m*1 structure at 0.25 GPa. KFe(MoO_4_)_2_ exhibits another reversible phase transition when the pressure is close to 1.3 GPa to, most probably, the *P*$\bar{3}$*c*1 phase [56]. In [47], it was found that the quasibinarity of the K_2_MoO_4_–Fe_2_(MoO_4_)_3_ system is broken as a result of the K_3_FeMo_4_O_15_ formation [57]. It is known that the double molybdates K_8_Hf(MoO_4_)_6_ and K_2_Hf(MoO_4_)_3_ are formed at the lateral side K_2_MoO_4_–Hf(MoO_4_)_2_ [58]. The double molybdates are not formed in the Fe_2_(MoO_4_)_3_–Hf(MoO_4_)_2_ system [59].

**Figure 1:**
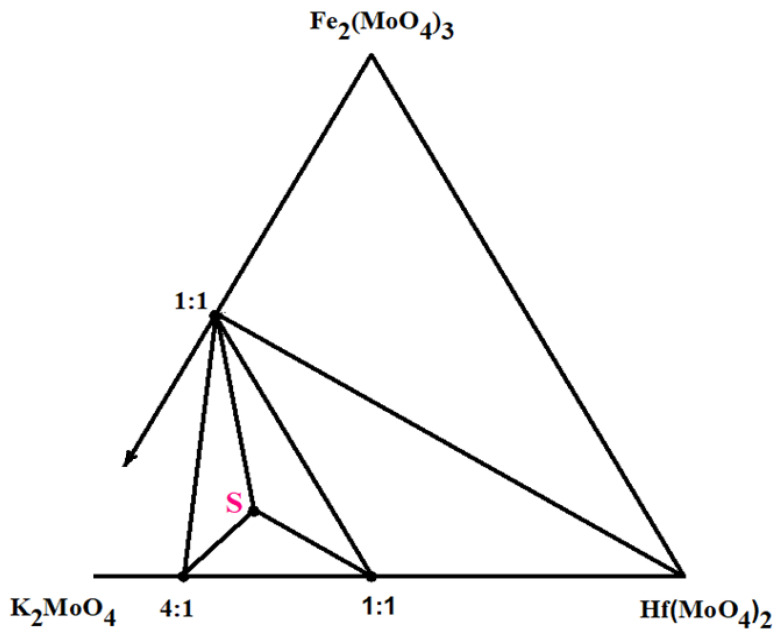
Subsolidus phase relations in the K_2_MoO_4_–Fe_2_(MoO_4_)_3_–Hf(MoO_4_)_2_ system at 823–873 K: **S**—K_5_FeHf(MoO_4_)_6_.

The powder diffraction data analysis of the annealed samples corresponding to several selected significant points of the K_2_MoO_4_–Fe_2_(MoO_4_)_3_–Hf(MoO_4_)_2_ system allowed us to determine the quasi-binary joins and to reveal the formation of a new triple molybdate. To prepare the samples of the selected compositions, the stoichiometric mixtures of the K_2_MoO_4_, Fe_2_(MoO_4_)_3_ and Hf(MoO_4_)_2_ molybdates were thoroughly ground in an agate mortar. The powder samples were annealed in the temperature range of 723–873 K for 150–200 h in the air. The single-phase K_5_FeHf(MoO_4_)_6_ sample was prepared by annealing the stoichiometric mixture of the simple molybdates at 873 K for 80–100 h. The phase equilibria of the K_2_MoO_4_–Fe_2_(MoO_4_)_3_–Hf(MoO_4_)_2_ system in the subsolidus region is shown in Figure 1. Six quasibinary cross-sections exist in the subsolidus region 823–873 K: KFe(MoO_4_)_2_–K_8_Hf(MoO_4_)_6_; KFe(MoO_4_)_2_–K_2_Hf(MoO_4_)_3_; KFe(MoO_4_)_2_–Hf(MoO_4_)_2_; KFe(MoO_4_)_2_–K_5_FeHf(MoO_4_)_6_; K_8_Hf(MoO_4_)_6_–K_5_FeHf(MoO_4_)_6_; K_2_Hf(MoO_4_)_3_–K_5_FeHf(MoO_4_)_6_. Thus, the formation of only one ternary molybdate was detected in the K_2_MoO_4_–Fe_2_(MoO_4_)_3_–Hf(MoO_4_)_2_ system.

### 2.2. Crystal Structure of K_5_FeHf(MoO_4_)_6_

Single crystals of K_5_FeHf(MoO_4_)_6_ suitable for a single crystal structure analysis were grown by the spontaneous flux crystallization with the use of superfluous reactant K_2_Mo_2_O_7_ as a flux. The K_5_FeHf(MoO_4_)_6_ powder was used as the charge. The weight ratio of the solvent to the charge was selected to be equal to 3:1. The starting mixture was ground in an agate mortar into a homogeneous powder for 15 min and put into a 20 mL quartz crucible with a smooth inner wall and a top cap. The batch was melted at 873 K and was held at this temperature for 7 h to reach a completely melting and transparent solution. After that, slow cooling was performed from 873 to 473 K at the rate of 2 K h^−1^. Then, the furnace was switched off and naturally cooled to room temperature. The heating of the ground mixture, the isothermal holding, and the slow cooling of the melt were controlled and kept automatically with the precision of ±0.5 K. Transparent brown and regular shaped crystals sized 1–2 mm were separated from the flux by breaking up the solidified cake.

The crystal structure of K_5_FeHf(MoO_4_)_6_ was determined by X-ray single crystal diffraction analysis. Centrosymmetrical space group *R*$\bar{3}$*c* (167) was chosen based on the analysis of the absences in the data array. The structure refinement showed the cooperative occupation of the Hf and Fe atom positions. The SOFs for the Hf and Fe atoms in positions M1 and M2 were refined from the difference electron density maps and fixed with ratio 0.732/0.268 for M1 (opposite for M2) in the final refinement to reduce the shifts. Position M1 is located on the inversion axis 3 and is occupied mainly by Hf atoms. Position M2 is located at the intersection point of axes 2 and 3 and is occupied mainly by Fe atoms.

**Figure 2:**
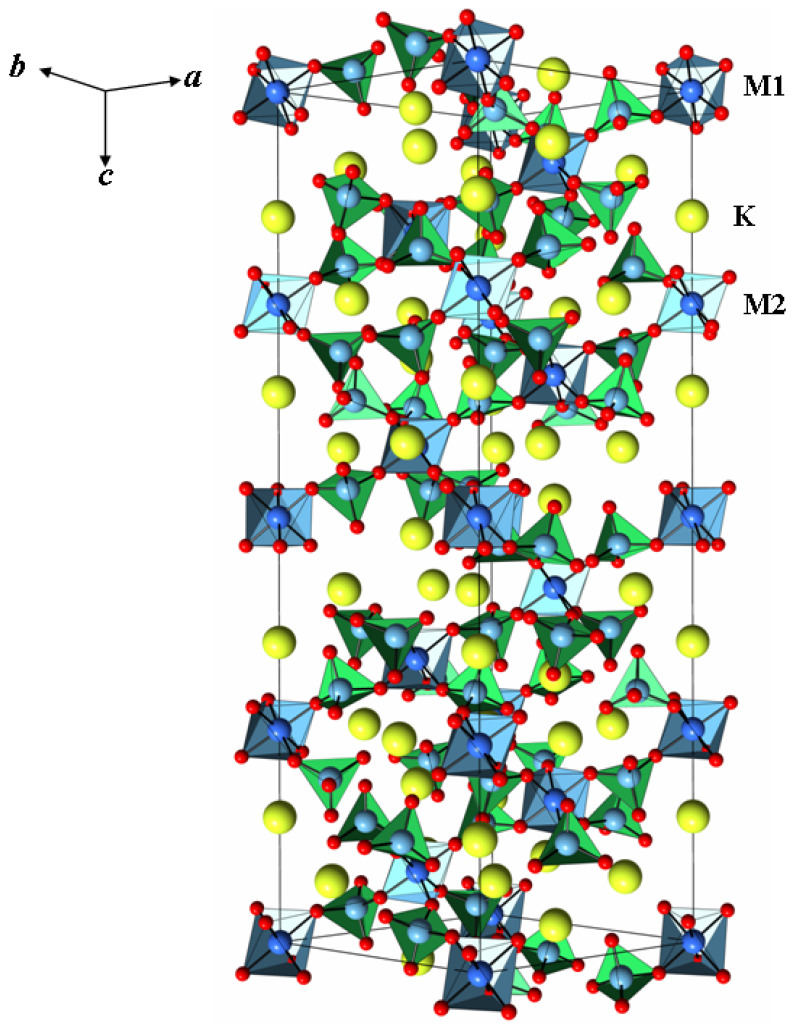
Fragment of the K_5_FeHf(MoO_4_)_6_ crystal structure. The unit cell is outlined. Lone atoms, excepting K, are omitted for clarity.

The obtained K_5_FeHf(MoO_4_)_6_ structure can be observed in Figure 2. The compound K_5_FeHf(MoO_4_)_6_ is isostructural to Tl_5_BiHf(MoO_4_)_6_ [21], K_5_InHf(MoO_4_)_6_ [23], K_5_LuHf(MoO_4_)_6_ [25], Cs_5_BiZr(MoO_4_)_6_ [27] and K_5_ScHf(MoO_4_)_6_ [31]. The crystal structure of K_5_FeHf(MoO_4_)_6_ is a three dimensional mixed-metal framework, which is built by a regular alternation of Mo tetrahedra and two types of (Fe,Hf)O_6_ octahedra, which are linked, one to another, via O-corner sharing (Figure 2). A molybdenum atom is located in the common site in the structure. It has a tetrahedral oxygen coordination environment with the range of Mo–O distances within 1.716(2)–1.801(2) Å and an average of 1.763(2) Å. This value is in agreement with the common Mo–O bond distance [36]. The Fe and Hf atoms, located statistically in the M1 and M2 positions, have an octahedral environment with M–O distances of 2.051(2) and 2.019(2) Å. It should be pointed out that cation disorder in the M1 and M2 positions is a characteristic of this structural type, as shown in Table S1. The alkaline K^+^ cation is located in two positions with the formation of a nine-vertex, with triplets of distances K(1)–O equal to 2.795(2) × 3, 2.843(2) × 3 and 3.143(2) × 3 Å, and twelve-vertex with three pairs of close distances (K2–O 2.766(2) × 2, 2.988(2) × 2 and 3.015(2) × 2 Å) and three pairs of longer distances (K2–O 3.278(2) × 2, 3.292(2) × 2 and 3.465(2) × 2 Å) (Table 2). Variations in these distances, arising from different coordination types of O atoms to K, Fe, and Hf cations, are commensurate to the distances observed in other structures of similar composition and type [23,25,27,31].

The main crystallographic data and refinement details are given in Table 1. The selected bond lengths in the K_5_FeHf(MoO_4_)_6_ structure are given in Table 2. The supplementary data CCDC 2,232,497 contain the supplementary crystallographic data for the K_5_FeHf(MoO_4_)_6_ compound. These data can be obtained free of charge via http://www.ccdc.cam.ac.uk/conts/retrieving.html (accessed on 10 December 2022), or from the Cambridge Crystallographic Data Centre, 12 Union Road, Cambridge CB2 1EZ, UK; Fax: (+44)1223-336-033; or e-mail: deposit@ccdc.cam.ac.uk.

**Table 1:** Crystal data and structure refinement details for K_5_FeHf(MoO_4_)_6_.

Empirical Formula	FeHfK_5_Mo_6_O_24_
Formula wt, g × mol^−1^	1389.48
Crystal system	trigonal
Space group	*R* $\bar{3}$ *c*
Crystal cell dimensions, Å	*a* = *b* = 10.4633(3)
	*c* = 37.3113(9)
Cell volume, Å^3^	3537.60(17)
Z	6
d_calc_, g/cm^3^	3.913
μ, mm^−1^	9.043
F(000)	3822
Crystal size, mm	0.06 × 0.05 × 0.04
θ range for data collection, deg.	2.50–27.49
Index ranges	−13 ≤ *h* ≤ 13 −13 ≤ *k* ≤ 13 −32 ≤ *l* ≤ 48
*I_hkl_* coll	8381
*I_hkl_* > 2*σ_I_* (*R*_int_)	908 (*R*_int_ = 0.0371)
GOOF for *F*^2^	1.099
*R* (*I* > 2*σ_I_*)	*R*_1_ = 0.0137, *wR*_2_ = 0.0317
*R* (*I_hkl_* coll)	*R*_1_ = 0.0145, *wR*_2_ = 0.0319
Largest diff peak, hole e/Å^3^	1.038 and −0.525

**Table 2:** Selected interatomic distances (d) for K_5_FeHf(MoO_4_)_6_.

Mo Tetrahedron		(Fe,Hf) Octahedra	
Bond	*d*, Å	Bond	*d*, Å
Mo(1)-O(1)	1.800(2)	M(1)–O(1)	2.051(2) × 6
Mo(1)-O(2)	1.801(2)	M(2)–O(2)	2.019(2) × 6
Mo(1)-O(3)	1.735(2)	K1 polyhedron	
Mo(1)-O(4)	1.716(2)	K(1)–O	2.795(2)–3.143(2)
〈Mo(1)-O〉	1.763(2)	K2 polyhedron	
		K(2)–O	2.766(2)–3.015(2)
			3.278(2)–3.292(2)

**Figure 3:**
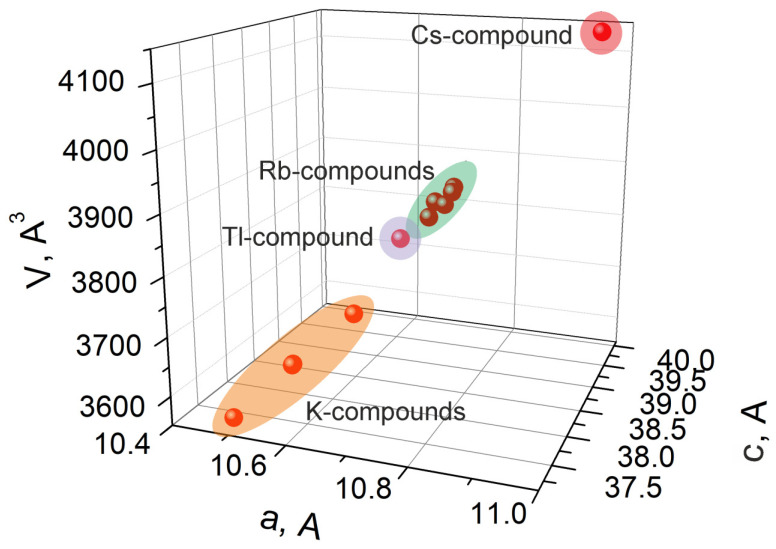
*A*_5_*MT*(MoO_4_)_6_ (*A* = K, Rb, Cs, Tl; *M* = In, Bi, *Ln*; *T* = Zr, Hf) crystals in the *a*–*c*-*V* space.

All of the *A*_5_*MT*(MoO_4_)_6_ compounds, the unit cell parameters of which are known, are listed in Table S1 and shown in Figure 3. As is evident from Table S1, the giant cell volume variation, by ~17%, is possible among the known members of the *A*_5_*MT*(MoO_4_)_6_ crystal family, and this indicates the high stability of this structure type. As is seen in Figure 3, in the *a*-*c*-*V* space, all known crystals can be captured by the extremely narrow elongated inclined ellipsoid, which apexes are determined by Cs_5_BiZr(MoO_4_)_6_ and K_5_FeHf(MoO_4_)_6_ points. The relatively stable *c*/*a* ratio is a characteristic feature of the *A*_5_*MT*(MoO_4_)_6_ crystal family, and, in the design of new compounds, the selection of *T*-*A* cation pairs should be carried out with keeping *c*/*a*~3.5–3.6.

**Figure 4:**
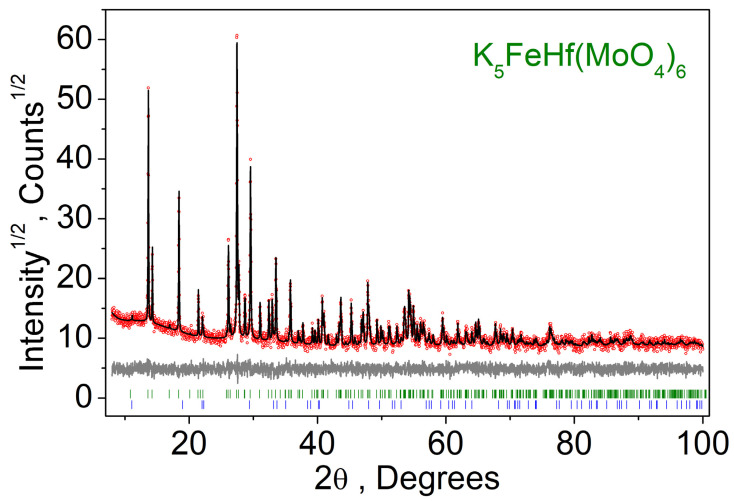
Measured (red), calculated (black) and differential (gray) X-ray diffraction patterns of the K_5_FeHf(MoO_4_)_6_ powder sample.

**Figure 5:**
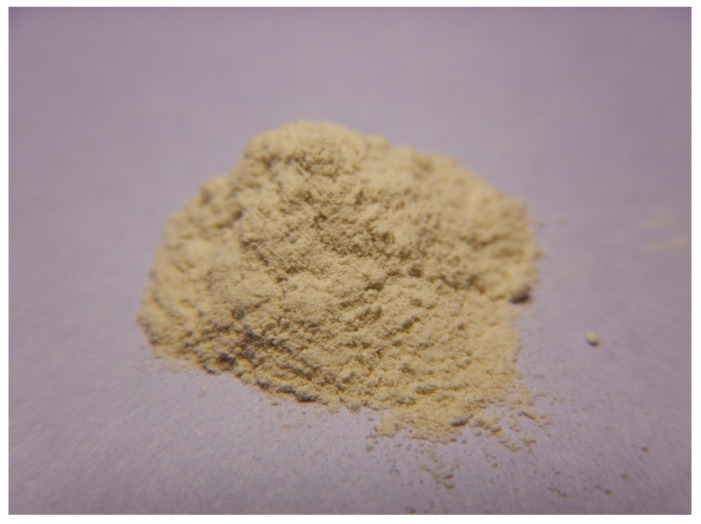
Digital photo of the K_5_FeHf(MoO_4_)_6_ powder.

The physical properties of the K_5_FeHf(MoO_4_)_6_ were measured with the use of the powder sample. The XRD pattern recorded for the synthesized powder sample of K_5_FeHf(MoO_4_)_6_ is presented in Figure 4. All of the peaks were indexed by trigonal cell (*R*$\bar{3}$*c*) with the parameters close to the single crystal data of K_5_FeHf(MoO_4_)_6_, as obtained in the present study. Therefore, this structure was taken as the starting model for the Rietveld refinement, which was performed using TOPAS 4.2 [60]. There are two Hf/Fe sites in the asymmetric part of the unit cell. The Hf:Fe ratio in each of these sites was refined in the assumption of 100% site occupation and the total Hf:Fe = 1:1 ratio in the final chemical formula. The refinement was stable and gave low R-factors (Table S2, Figure 4). A digital photo of the K_5_FeHf(MoO_4_)_6_ powder sample is presented in Figure 5. The sample is characterized by a light brown tint attributed to the presence of Fe^3+^ ions.

### 2.3. Thermochemical Properties

**Figure 6:**
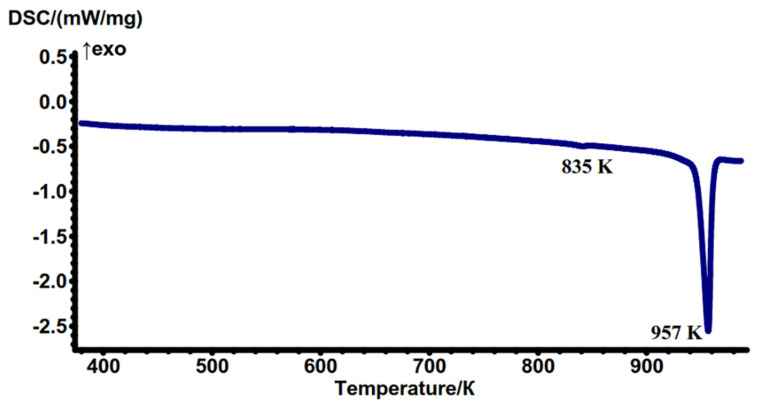
DSC curve obtained on the K_5_FeHf(MoO_4_)_6_ powder.

The thermal stability of K_5_FeHf(MoO_4_)_6_ was characterized using DSC, as shown in Figure 6. From the DSC curve, there are two endothermal peaks, located at 835 and 957 K. The small endothermic peak at 835 K has a phase transition enthalpy of ΔH = −0.45 J/g. A sharp peak at the temperature of 957 K corresponds to the incongruent melting of the sample (ΔH = −101 J/g). For comparison, the known thermal parameters of the *A*_5_*MT*(MoO_4_)_6_ crystals are listed in Table S3 [21,31,33,34,35,61]. As it is seen in Table S3, the K_5_FeHf(MoO_4_)_6_ melting point is among the typical values observed for *A*_5_*MT*(MoO_4_)_6_ crystals. The analysis of the powder XRD pattern of the solidified melt revealed that K_5_FeHf(MoO_4_)_6_ is decomposed to double and simple molybdates, and it indicates that K_5_FeHf(MoO_4_)_6_ is an incongruent melting compound.

### 2.4. Vibration Dynamics

**Figure 7:**
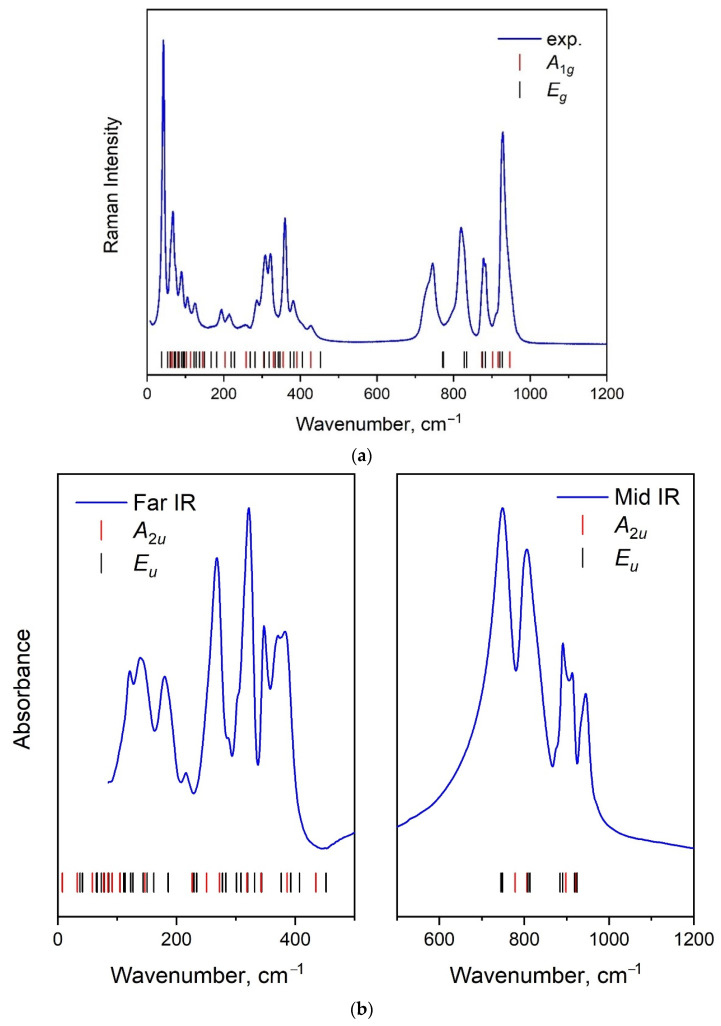
Raman (**a**) and infrared (**b**) spectra of K_5_FeHf(MoO_4_)_6_.

The Raman and infrared spectra collected for K_5_FeHf(MoO_4_)_6_ at normal conditions are shown in Figure 7. In both cases, the phonon modes are observed up to 1000 cm^−1^, with a gap from approximately 450 to 700 cm^−1^. The existence of such a phonon gap is typical of molybdate crystals with MoO_4_ tetrahedra [11,21,62,63,64,65]. According to the group theory, the vibrational modes of K_5_FeHf(MoO_4_)_6_ in the center of the Brillouin zone can be listed as Γ_vibr_ = 17*A*_1*g*_ + 19*A*_2*g*_ + 36*E_g_* + 18*A*_1*u*_ + 20*A*_2*u*_ + 38*E*_u_, where *A*_1*g*_ and *E_g_* modes are Raman-active, *A*_1*u*_ and *A*_2*g*_ modes are silent, *A*_2*u*_ and *E_u_* modes are infrared-active. The acoustic modes are Γ_acoustic_ = *A*_2*u*_ + *E_u_*. According to the structural data presented above, the K_5_FeHf(MoO_4_)_6_ structure contains one crystallographically independent MoO_4_ tetrahedron. The [MoO_4_]^2−^ ion is of *T_d_* symmetry and has four internal vibration modes: ν_1_—symmetric stretching, ν_3_—asymmetric stretching, ν_2_ and ν_4_—symmetric and asymmetric bending modes, respectively. The correlation diagram for the internal MoO_4_ vibrations in the K_5_FeHf(MoO_4_)_6_ crystal is given in Table 3. According to Table 3, it can be concluded that three Raman-active and three infrared-active modes exist for MoO_4_ ν_1_ vibrations. In the Raman spectrum, the ν_3_ modes are 3*A*_1*g*_ + 6*E_g_*, and, in the infrared spectra, they are 3*A*_2*u*_ + 6*E_u_*. Thus, 12 vibrational bands should be present in the Raman, as in infrared spectra, between 700 and 1000 cm^−1^. The classification of the spectral bands according to irreducible representations is extremely difficult in the case of unpolarized spectra and, as can be seen in Figure 7, these bands are strongly overlapped. For comparison, the calculated DFT wavenumbers of the Raman and infrared modes are shown in Figure 7, with vertical tick marks with the corresponding irreducible representations labels. The existence of the phonon gap between 450 and 700 cm^−1^ is confirmed by the calculations and a large number of modes below 450 cm^−1^ are observed. Fifteen of these modes are related to ν_2_ and ν_4_ types and observed in the Raman and infrared spectra from 270 to 450 cm^−1^. The group of lines are rotation-like vibrations of MoO_4_ tetrahedra. The bands below 175 cm^−1^ are the lattice modes of K_5_FeHf(MoO_4_)_6_.

**Figure 8:**
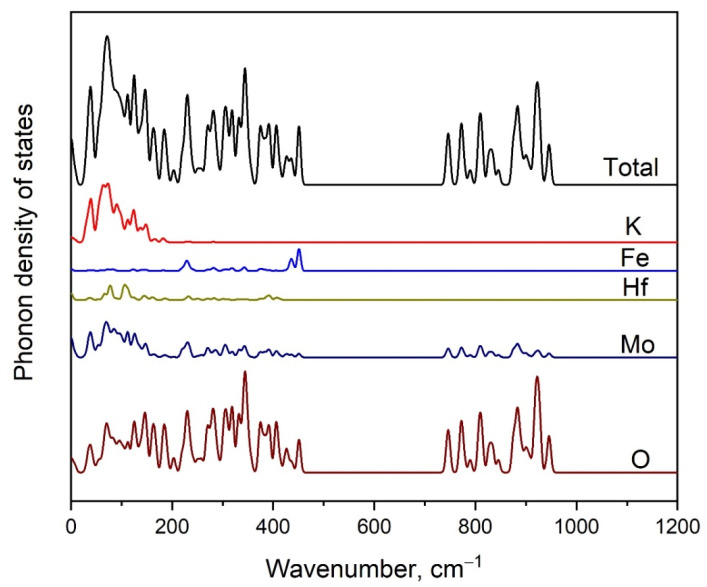
Calculated phonon density of states in K_5_FeHf(MoO_4_)_6_.

In Figure 8, we show the partial phonon density of states for K_5_FeHf(MoO_4_)_6_. The high-wavenumber part is dominated by the vibrations of oxygen atoms in the MoO_4_ tetrahedra (ν_3_ and ν_1_ modes). It is interesting to note that the O ions are involved in almost all of the modes that appeared in the low-wavenumber region. As was mentioned above, all types of structural units yield vibrations in the region of spectra below 175 cm^−1^. Several peaks related to the vibrations of Fe atoms are observed at 228, 435 and 450 cm^−1^.

### 2.5. Calculated Electronic Structure

**Figure 9:**
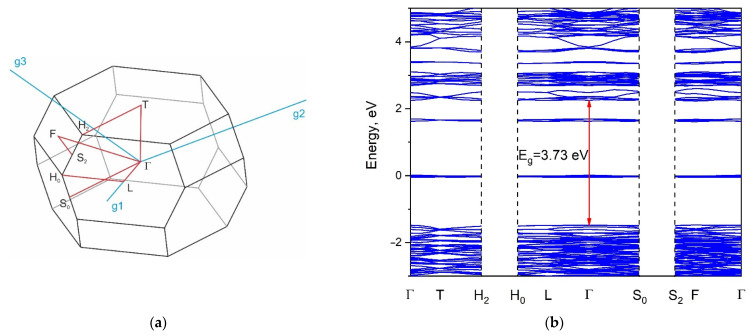
Brillouin zone (**a**) and band structure (**b**) of K_5_FeHf(MoO_4_)_6_.

**Figure 10:**
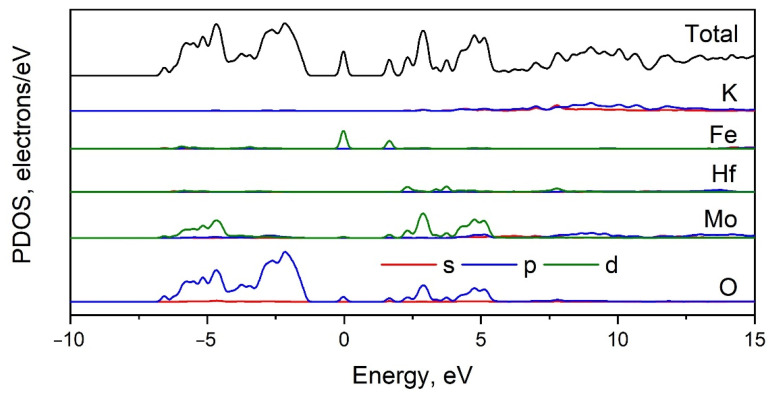
Total and partial electronic DOS of K_5_FeHf(MoO_4_)_6_.

The Brillouin zone (BZ) and calculated electronic band structure of K_5_FeHf(MoO_4_)_6_ are shown in Figure 9a,b, respectively. The list of *k*-points defining the path along the Brillouin zone should be written as Γ-T-H_2_|H_0_-L-Γ-S_0_|S_2_-F-Γ. The coordinates of these points are Γ(0,0,0), T(0.5,0.5,0.5), H_2_(0.794,0.206,0.5), H_0_(0.5,−0.206,0.206), L(0.5,0,0), S_0_(0.353,−0.353,0), S_2_(0.647,0,0.353). The conduction band minimum and the valence band maximum are located in the center of the BZ, and the calculated direct band gap is equal to 3.73 eV. The obtained band gap value is typical of crystals with MoO_4_ tetrahedra [65]. As can be seen in Figure 10, the valence band maximum is dominated by the O-2*p* states, while the conduction band minimum is formed by Mo-4*d* states. Thus, we can say that the optical transitions in K_5_FeHf(MoO_4_)_6_ are associated with the charge transfer from oxygen to molybdenum ions. Some weak bands are observed in the electronic density of states (DOS), shown in Figure 10, for Fe and Hf ions, and these peaks are related to the narrow flat branches that appeared in the band structure at 1.48 and 3.14 eV above the valence band top (Figure 9b). The low-lying unoccupied levels predicted for Fe are, in fact, avatars of excited Fe 3*d* states that cannot be correctly calculated because the software used does not allow a switch on of the interaction between electrons within the 3*d* shell. However, the spectral measurements described in the next section show that the d-d transitions are really the lowest electronic excitations in the crystal under study, and the corresponding spectra look very similar to those for the isolated Fe^3+^ ion, which is in agreement with the band structure calculations.

### 2.6. Reflection Spectrum and Experimental Bandgap

The crystal structure of K_5_FeHf(MoO_4_)_6_ belongs to the trigonal symmetry class and is of the *R*$\bar{3}$*c* space symmetry group. The Fe^3+^ ions are positioned within the oxygen octahedra and occupy two inequivalent positions with local symmetry C_3i_ (occupancy 0.268) and D_3_ (occupancy 0.732), respectively, whilst the rest of the occupancies belong to hafnium. The deviation from the undistorted O_h_ symmetry in both cases is rather minor, and the lower crystal field symmetry components are expected to be rather weak.

**Figure 11:**
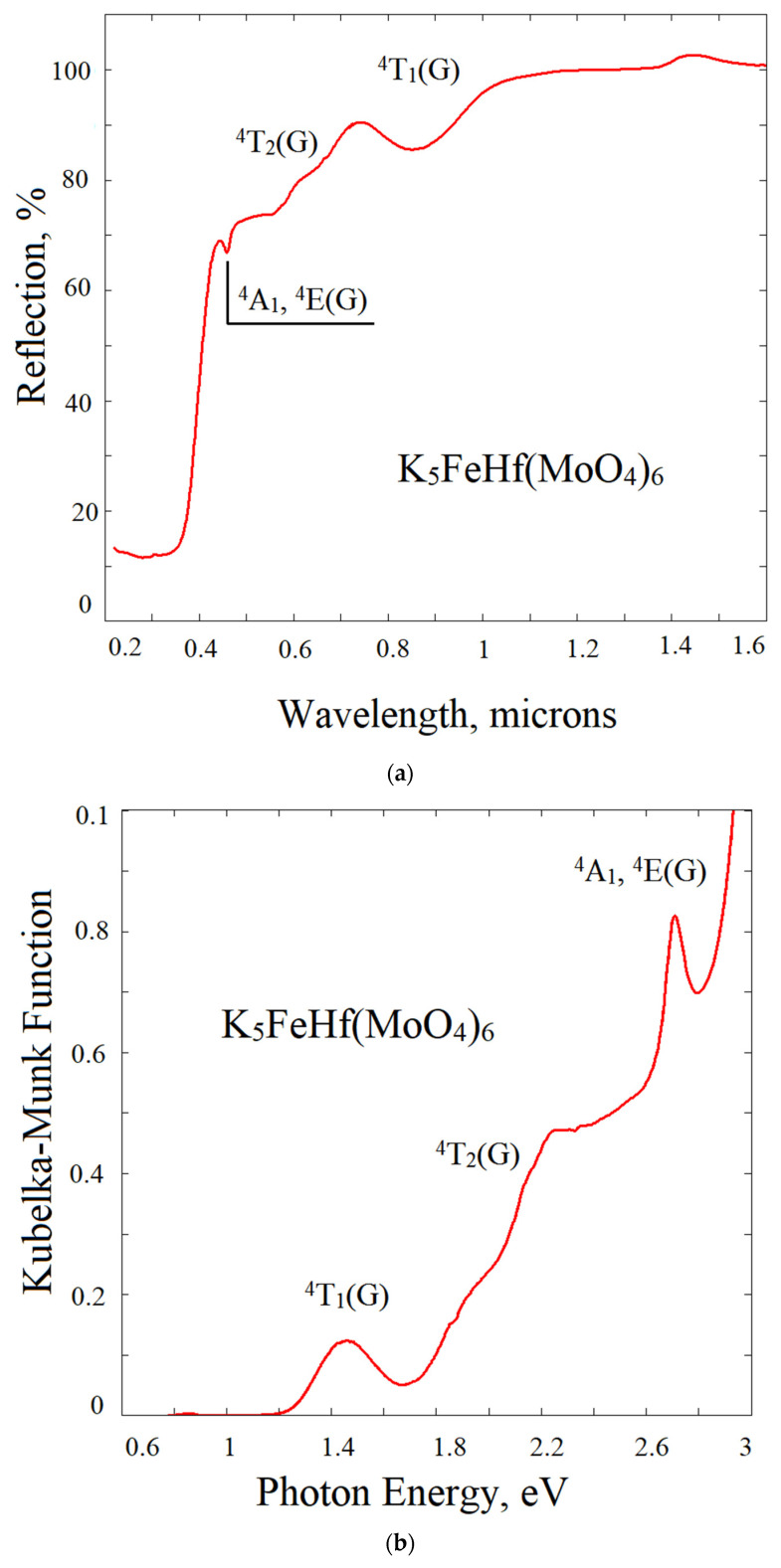

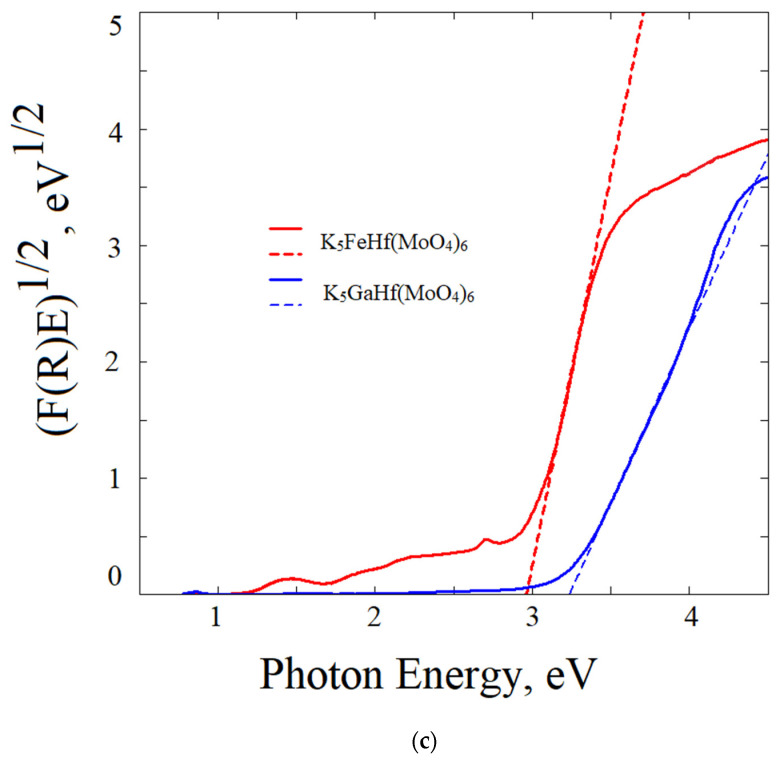
(**a**) Diffuse reflection spectrum of K_5_FeHf(MoO_4_)_6_. (**b**) Kubelka-Munk function of K_5_FeHf(MoO_4_)_6_. (**c**) Determination of indirect bandgaps of K_5_FeHf(MoO_4_)_6_ and K_5_FGaHf(MoO_4_)_6_.

In Figure 11a, the diffuse reflection spectrum of K_5_FeHf(MoO_4_)_6_ is shown; the low energy part of the Kubelka-Munk function F(R) = (1 − R)^2^/(2R), where R is the reflection coefficient, is shown in Figure 11b. The spectral features observed in the 1–3 eV range are characteristic for the *d^5^* electronic system and are well assignable to the transitions from the Fe^3+^ ground state ^6^A_1_ to ^4^T_1_(G) ^4^T_2_(G), and ^4^A_1_, ^4^E(G) states. The corresponding energy for the ^4^T_1_(G) state is 1.46 eV, and the narrow peak corresponding to the degenerate ^4^A_1_ and ^4^E states is observed at 2.71 eV. In the intermediate energy range, three subbands can be located at 1.83, 2.13 and 2.25 eV; these subbands must be ascribed to the ^4^T_2_(G) states at two inequivalent sites within the crystal structure, rather than to the splitting of the ^4^T_2_(G) state by the low symmetry crystal field components.

The direct and indirect bandgaps were determined using the modified Kubelka-Munk functions. The fundamental absorption edge for the indirect transition in K_5_FeHf(MoO_4_)_6_ (Figure 11c, red line) is found to be more pronounced than that of the direct transition. The indirect bandgap value is found to be 2.97 eV, while the direct bandgap in K_5_FeHf(MoO_4_)_6_ is equal to 3.21 eV. Remarkably, these bandgap values are noticeably lower than those known for a variety of molybdates. The latter are typically assigned to the transitions between the bands originating from the energy levels of the MoO_4_ group. The corresponding bandgap in molybdates may be due to a charge transfer band in the system of Mo^6+^—O^2−^ and is expected to be observed at 3.5–4 eV; additionally, this was admitted by the calculations presented in Section 2.5. of the present paper. The bandgap narrowing in K_5_FeHf(MoO_4_)_6_, with respect to the common molybdates, cannot be ascribed to the contribution of subbands originating from the Hf^4+^ ions because the transitions originating from the Hf subsystem in the oxides are expected in the range of 5 eV.

To clarify the origin of the bandgap narrowing, we synthesized the reference crystal with the K_5_GaHf(MoO_4_)_6_ content in which Fe^3+^ ions are replaced by Ga^3+^ ions, featured by the closest ionic radius to Fe^3+^ and by the closed shell electron configuration 1*s*^2^2*s*^2^2*p*^6^3*s*^2^3*p*^6^3*d*^10^, which is different from the opened shell *3d^5^* configuration of Fe^3+^. The optical transitions starting from the closed *d* shell are known to be substantially higher in energy than those from the opened *d* shell. The obtained K_5_GaHf(MoO_4_)_6_ compound, as expected, is characterized by a crystal structure very similar to K_5_FeHf(MoO_4_)_6_ and belongs to the same space group *R*$\bar{3}$*c*, with the cell parameters coinciding those of K_5_FeHf(MoO_4_)_6_ within 0.1%. The Kubelka-Munk function built using the diffuse reflectance data modified for the case of indirect transition is plotted in Figure 11c by the blue line. The indirect bandgap is equal to 3.24 eV, while the direct bandgap for K_5_GaHf(MoO_4_)_6_ is as wide as 3.95 eV. Therefore, both the direct and indirect K_5_GaHf(MoO_4_)_6_ bandgaps are substantially larger than those of K_5_FeHf(MoO_4_)_6_ and narrowing the bandgap of K_5_FeHf(MoO_4_)_6_ must be associated with an additional absorption in the Fe^3+^ system. Three of the five forbidden *d*-*d* transitions of Fe^3+^ are observed in the range below 3 eV, and the next two *d*-*d* transitions (to ^4^T_2_(D) and ^2^E(D) states) can lie in the region above 5 eV; however, their absorption must be of the same order of magnitude, such as that of lower states, and ^4^T_2_(D) and ^2^E(D) cannot contribute to the onset of the fundamental absorption edge.

The most direct hypothesis for the additional strong absorption of K_5_FeHf(MoO_4_)_6_ in the range between 3 and 4 eV is the charge transfer transition O^2−^ 2*p*—Fe^3+^ 3*d*. However, the known charge transfer band positions of the Fe^3+^ ions in oxides are typically in the range 4.4–4.8 eV (see, e.g., references in [66]). In the present situation, the only eligible explanation for the bandgap narrowing is the absorption at the Mott-Hubbard optical excitation channel (see, e.g., [67]). Mott-Hubbard optical transitions are interatomic *d*-*d* excitations. These excitations are shown to be the reason for the bandgap narrowing of gadolinium iron huntite [66]. The factor that favors interatomic excitations in the Fe^3+^ system is the antiferromagnetic interaction that is known in huntites. We showed (Section 2.8) that the antiferromagnetic interaction is also present in K_5_FeHf(MoO_4_)_6_. Surprisingly, in K_5_FeHf(MoO_4_)_6_, Mott-Hubbard excitation in the Fe^3+^ system is observed at the rather large distance of 7 Å between the Fe^3+^ sites within the crystal lattice, as well as in the presence of structural Fe/Hf disorder.

### 2.7. Electrical Properties

**Figure 12:**
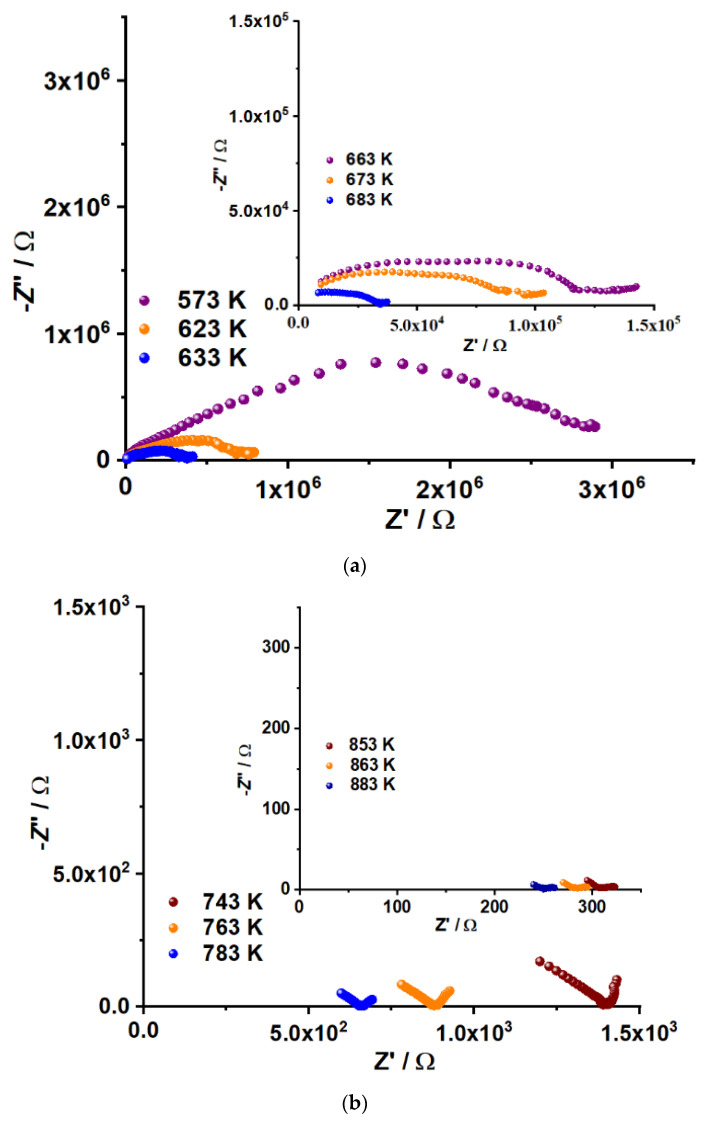
Nyquist plots of K_5_FeHf(MoO_4_)_6_ at different temperatures. (**a**) 573–683 K, (**b**) 743–883 K.

The electrical properties of K_5_FeHf(MoO_4_)_6_ were investigated by impedance spectroscopy. This is a reliable technique to study the electrical properties of the oxide compounds [21,67,68,69,70,71,72,73,74]. The Nyquist plots (Z″ vs. Z′), recorded for the K_5_FeHf(MoO_4_)_6_ sample at different temperatures, are shown in Figure 12. The spectra were recorded in the frequency range between 1 Hz and 1 MHz. Commonly, the impedance diagram of a typical electrolyte consists of three semicircles, and each semicircle represents a distinct process. The high frequency semicircle and medium frequency semicircle represent the contributions of the grain bulk and grain boundary, while the low frequency semicircle represents the electrode contribution (C = 10^−4^−10^−5^ F). In practice, not all characteristic impedance arcs can be observed in the experimental spectrum. In our experiment, the capacitance values at the level of 10^−10^ F were observed for the first semicircle, which can be considered as the average values of the capacitances for the bulk and grain boundary conductivities (10^−12^ and 10^−8^ F). Thus, the semicircle in the −Z″ vs. Z′ plots is associated with the sum of these two contributions. Moreover, at a certain temperature, a straight line appears just after the depressed semicircle. This tail is due to the effect of the electrode processes. It can be seen that the feature of impedance is varied at different temperatures. The resistance decreases with the increasing temperature and the conductivity increases accordingly. This behavior is a characteristic of solid electrolytes, and it is in a good agreement with the results reported previously for selected oxides [21,72,73,74].

**Figure 13:**
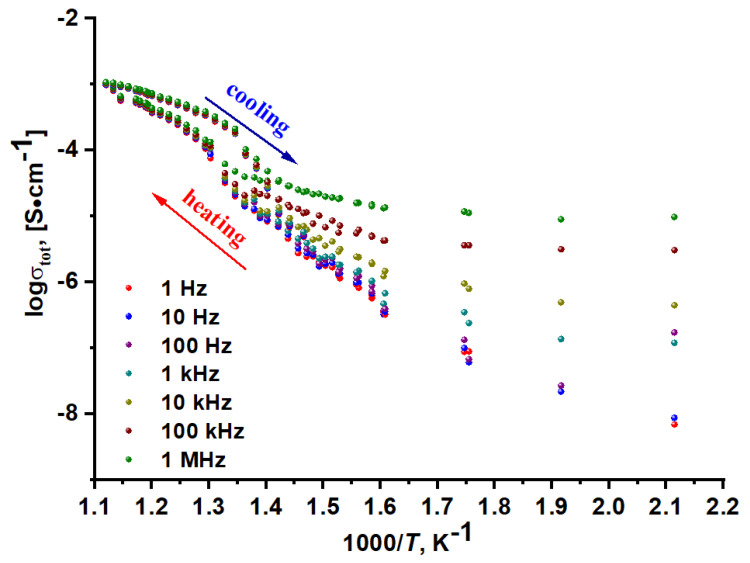
K_5_FeHf(MoO_4_)_6_ conductivity as a function of 1000/T at different frequencies.

In Figure 13, the variation in the ac conductivity (*σ*) with 1000/T at various frequencies is presented for K_5_FeHf(MoO_4_)_6_. The conductivity dependences have two linear parts, with different slopes. A change of the graph in the slope is observed near the transition temperature of approximately 750 K. Below and above the phase transition, the dependences can be described by the Arrhenius–Frenkel law, i.e., the observed processes are thermally activated. This behavior is typical of conductivity in most solid electrolytes, and it may be considered as an indication of the ionic transport in K_5_FeHf(MoO_4_)_6_. Molybdate K_5_FeHf(MoO_4_)_6_ has a comparatively low conductivity (2.9 × 10^−5^ S cm^−1^ at 550 K), and, as a result of phase transition, it passes into the superionic state (about 10^−3^ S cm^−1^ at 850 K). In the high-temperature range (740–893 K, cooling mode), the activation energy for the electrical conduction is ~0.8 eV and the electrical conductivity reaches 4 × 10^−4^ S/cm at 773 K. Then, at 893 K, it reaches a value as high as 1.1 × 10^−3^ S cm^−1^. The K_5_FeHf(MoO_4_)_6_ electrical conductivity values are slightly lower than those of K_5_CrHf(MoO_4_)_6_, but higher than those of K_5_ScHf(MoO_4_)_6_ and K_5_InHf(MoO_4_)_6_ (Table 4). It is seen in Table 4 that the replacement of the trivalent element with iron did not significantly improve the electrical conductivity of the compound, but, at the same time, the influence of the trivalent cation nature is observed.

At high temperatures, the conductivity does not significantly depend on the frequency. At the temperatures below 700 K (Figure 13), the conductivity data show the behavior is strongly dependent on the frequency. It is seen that the K_5_FeHf(MoO_4_)_6_ conductivity gradually increases with the increase in the applied electric field frequency. The high K_5_FeHf(MoO_4_)_6_ conductivity is possible due to sufficiently large sizes of voids in the structure, which appreciably decrease the steric hindrances to the ion transport. The crystal-chemistry analysis of K_5_FeHf(MoO_4_)_6_ shows that the Fe^3+^, Hf^4+^ and Mo^6+^ cations, which are part of the crystal framework, form stronger chemical bonds with oxygen than the singly charged K^+^ cations located in structural channels. This indicates a higher mobility of K^+^ cations in comparison with the “framework” cations, Fe^3+^, Hf^4+^ and Mo^6+^.

### 2.8. Magnetic Properties

**Figure 14:**
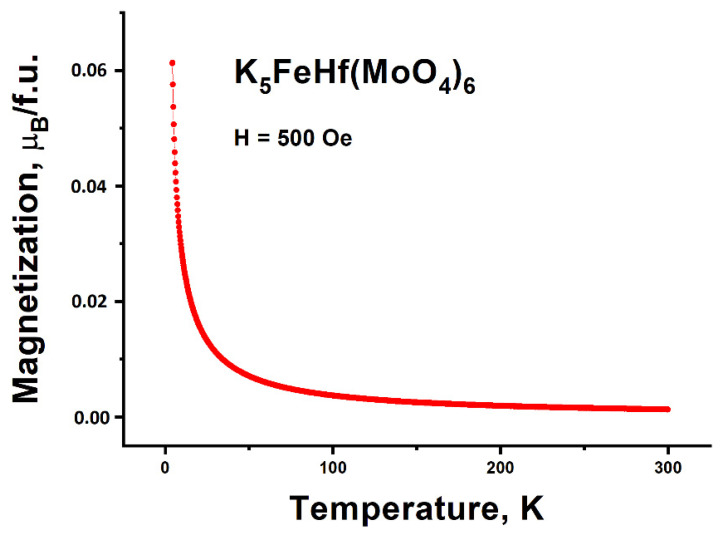
Temperature dependence of magnetization measured in the 500 Oe magnetic field.

The temperature dependence of K_5_FeHf(MoO_4_)_6_ magnetization in the 500 Oe magnetic field is presented in Figure 14; this dependence evidences the paramagnetic state of K_5_FeHf(MoO_4_)_6_ down to 4.2 K. At low temperatures, in the vicinity of 4.2 K, the tendency of magnetic moment value saturation can be noticed, and that is a sequence of the orientation nature of K_5_FeHf(MoO_4_)_6_ paramagnetism.

**Figure 15:**
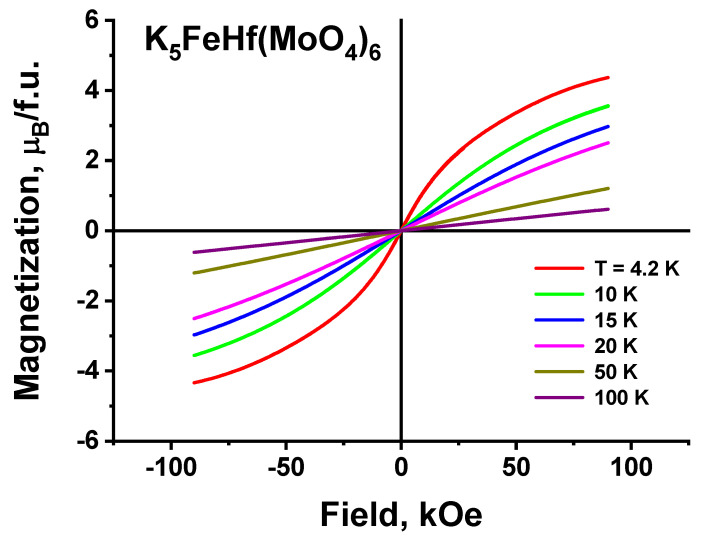
Field dependences of magnetization at different temperatures.

In Figure 15, the field dependences of magnetization are presented, as recorded at different temperatures. These curves evidence that, at all temperatures, including the liquid helium temperature (T = 4.2 K), the coercive force is equal to zero. This indicates the absence of the magnetic domains that are present in magnetically ordered crystals. Therefore, the field dependences of M(H) also confirm the statement that the crystal under study is a paramagnet in a good approximation. The preservation of the paramagnetic state is associated with large distances between the magnetic ions of iron, which leads to a small exchange integral value comparable to the dipole–dipole interaction. In this case, the magnetic ordering can be expected only at temperatures below 1 K.

**Figure 16:**
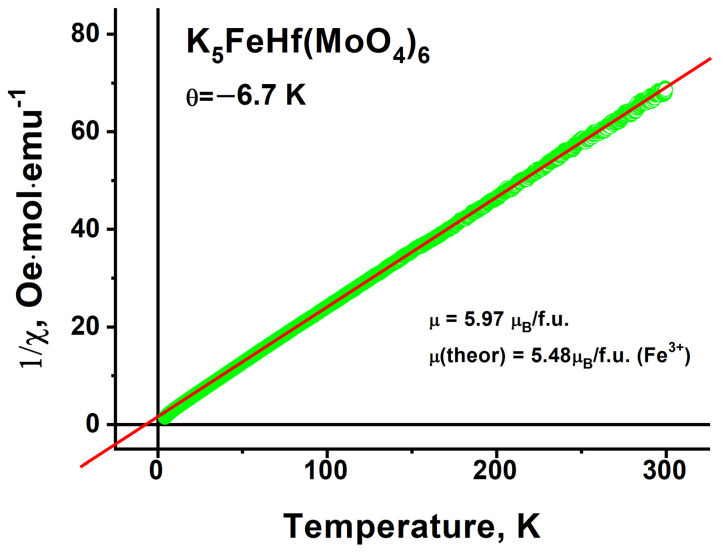
Dependence of inverse magnetic susceptibility measured at 500 Oe on the temperature.

The paramagnetic Curie temperature θ determined from the temperature dependence of the inverse susceptibility (Figure 16) is equal to θ = −6.7 K. This Curie temperature value indicates the presence of a weak antiferromagnetic interaction in the Fe subsystem of K_5_FeHf(MoO_4_)_6_. The effective magnetic moment corresponding to the Curie temperature value is equal to μ_ef_ = 5.97 μ_B_. Assuming that only Fe^3+^ ions possess a magnetic moment among all of the ions in a crystal, the theoretical value of the magnetic moment per formula unit equals μ^theor^ = g_s_·μ_B_·(S(S + 1))^1/2^, where g_s_ = 2 is the degeneracy factor accounting only the spin contribution to the magnetic moment, and S = 5/2 is the Fe^3+^ ion spin. The corresponding theoretical value μ^theor^ = 5.48 μ_B_ is in good agreement with the experimental one.

As we see, K_5_FeHf(MoO_4_)_6_ crystals preserve their paramagnetic state in the temperature range down to 4.2 K, in contrast to some other double molybdates, such as CsFe_5_(MoO_4_)_7_ [75] or RbFe_5_(MoO_4_)_7_ [22], which exhibit their magnetic ordering below 20 K. The paramagnetic Curie temperatures in those two compounds are two times larger than in K_5_FeHf(MoO_4_)_6_, and, hypothetically, the magnetic ordering in K_5_FeHf(MoO_4_)_6_ could be expected in the vicinity of 10 K, and that was not found. Most likely, this is due to the peculiarities of the magnetic structure in our crystal.

## 3. Materials and Methods

Commercially available Fe_2_O_3_ (chemically pure, Ural Plant of Chemical Reagents, Russia), HfO_2_ (chemically pure, IGIC RAS, Russia) and MoO_3_ (chemically pure, Red Chemist, Russia) were used as the starting materials for the synthesis of simple molybdates. Potassium molybdate K_2_MoO_4_ (pure) was supplied by Red Chemist, Russia. Iron molybdate Fe_2_(MoO_4_)_3_ and hafnium molybdate Hf(MoO_4_)_2_ were prepared at 673–1023 K for 120 h. First, the simple oxides were well mixed according to the nominal compositions of Fe_2_(MoO_4_)_3_ and Hf(MoO_4_)_2_ and ground in an agate mortar. For better reactivity, the reaction mixtures were then progressively calcined at 673–1023 K, and intervened by mixing and grinding every 24 h of annealing. In the synthesis, after every 24 h of annealing, the reaction cakes were cooled, grinded and mixed for better reactivity. The phase purity of the final products was verified by the XRD analysis in reference to the known structures of Fe_2_(MoO4)_3_ (01-083-1701 [76]) and Hf(MoO_4_)_2_ (00-038-1467 [77]).

Simple K_2_MoO_4_, Fe_2_(MoO_4_)_3_ and Hf(MoO_4_)_2_ molybdates were used in the study of the phase formation in the quasi-ternary system K_2_MoO_4_–Fe_2_(MoO_4_)_3_–Hf(MoO_4_)_2_. In addition, these simple molybdates were applied for the preparation of powder samples and the single crystal growth of K_5_FeHf(MoO_4_)_6_.

Powder X-ray diffraction measurements were carried out with the use of a Bruker D8 ADVANCE diffractometer (CuK_α_ radiation, Vantec-1 detector, maximum angle 2*θ* = 100°). The step size of 2*θ* was 0.0208°, and the counting time was 2 s per step. The XRD patterns were recorded at room temperature and normal atmospheric pressure. To identify the sample phase composition, the ICDD database was used.

The single-crystal X-ray diffraction data for K_5_FeHf(MoO_4_)_6_ (**I**) were collected on a Bruker Nonius X8 Apex diffractometer equipped with the graphite monochromated MoK*_α_* (*λ* = 0.71073 Å) radiation at 293(2) K. The *φ*- and *ω*-scan technique was employed to measure the intensities. Absorption corrections were applied empirically using the SADABS program [78]. The structure was solved by the direct methods of the difference Fourier synthesis and further refined by the full-matrix least squares method using the SHELXTL package [79]. The atomic thermal parameters for all of the atoms were refined anisotropically.

Differential scanning calorimetric (DSC) measurements were performed on an NETZSCH STA 449C TG/DSC/DTA (Jupiter) thermoanalyzer over the temperature range of 373–1000 K with the heating/cooling rates of 10 K min^−1^ Ar flow. Pt crucibles were used as vessels. Pt–PtRh thermocouples were applied for the temperature control. The temperature measurement precision was ±1 K. The DSC curves were calculated using a specially developed program from Netzsch. The Raman spectra were recorded with the help of the Horiba Jobin Yvon T64000 spectrophotometer using the excitation from the Spectra Physics Excelsior 532 nm laser. The spectral resolution of the Raman spectra was 2.7 cm^−1^. The infrared spectra were recorded with the help of the Bruker VERTEX 80 FTIR spectrometer.

The electrical conductivity was measured by the two-contact impedance spectroscopy method as a function of the temperature and frequency exploring an impedance meter Z-1500J. The measurements were performed in the heating/cooling mode in the frequency range of 1 Hz–1 MHz. The pure phase powder of K_5_FeHf(MoO_4_)_6_ was used for the tablet fabrication. The tablets with the thickness of 2 mm and 10 mm in diameter were prepared with the use of a hydraulic laboratory press PLG-12 at 100 bar and then sintered at 773 K for 2 h. For the making of electrodes, large disk surfaces were covered with a paste-type mixture of hexachloroplatinate (IV) ammonium (NH_4_)_2_[PtCl_6_] in toluene. Then, the tablet with the paste layers was annealed at 773 K for 1 h. The electrical conductivity *σ*_total_ for each fixed temperature was calculated by relation:

*σ*_total_ = *L*/*R*_total_ × *S*, (1)

where *σ*_total_, *L*, *S* and *R*_total_ are the total conductivity, thickness of specimen, area of the big tablet surface and the total resistance, respectively. The magnetic properties of K_5_FeHf(MoO_4_)_6_ were investigated using vibrating magnetometer Quantum Design PPMS (Krasnoyarsk Regional Center of Research Equipment of Federal Research Center “Krasnoyarsk Science Center SB RAS”) over the 4.2–300 K temperature range at magnetic fields up to 9 T.

## 4. Computation Details

The ab initio calculations were carried out using the density function theory (DFT) approach implemented within the CASTEP code [80]. The Vanderbilt-type ultra-soft pseudopotentials were used, and the energy cutoff was taken as 450 eV. The 3*s*^2^3*p*^6^4*s*^1^, 3*d*^5^4*s*^2^, 5*d*^2^6*s*^2^, 4*s*^2^4*p*^6^4*d*^5^5*s*^1^, 2*s*^2^2*p*^4^ valence electron configurations were used for K, Fe, Hf, Mo and O, respectively. The model structure of K_5_FeHf(MoO_4_)_6_ was fully optimized using the generalized gradient approximation (GGA) with the PBESol (Perdew-Burke-Ernzerhof for solids) exchange-correlation functional [81]. As a starting point, the structural parameters were used, as obtained in the present work. The convergence tolerance was set to 0.005 eV/Å and 0.02 GPa for the maximum force and maximum stress, correspondingly. The 3 × 3 × 3 Monkhorst-Pack mesh [82] was used for the Brillouin zone sampling.

## 5. Conclusions

A new, structurally disordered molybdate K_5_FeHf(MoO_4_)_6_ was synthesized and found to crystallize in the trigonal space group *R*$\bar{3}$*c*. The direct band gap value obtained from the first-principles calculations is 3.73 eV. According to the results of the lattice dynamics density functional theory calculations and the group theory analysis, most of the spectral lines above 175 cm^−1^ in the vibrational spectra of K_5_FeHf(MoO_4_)_6_ are associated with the MoO_4_ vibrations. The calculations of the partial phonon density of states revealed that the translations of oxygen ions involved almost all of the vibrational modes. K_5_FeHf(MoO_4_)_6_ is a paramagnetic down to 4.2 K. The negative Curie–Weiss temperature of −6.7 K indicates dominant antiferromagnetic interactions in the compound. The direct and indirect optical bandgaps of K_5_FeHf(MoO_4_)_6_ are 3.21 and 2.97 eV. The bandgap narrowing, with respect to a variety of molybdates and to the ab initio calculations, is explained by the Mott-Hubbard interatomic optical excitation channel.

## Figures and Tables

**Table 3:** Correlation diagram of MoO_4_ internal vibrations in K_5_FeHf(MoO_4_)_6_.

Molecular Group Symmetry.		Site Symmetry.		Space Group Symmetry
*T_d_*		*C*_1_		*D*_3*d*_
ν_1_, *A*_1_	→	*A*	→	*A*_1*g*_ + *A*_1*u*_ + *A*_2*g*_ + *A*_2*u*_ + 2*E_g_* + 2*E_u_*
ν_2_, *E*	→	2*A*	→	2*A*_1*g*_ + 2*A*_1*u*_ + 2*A*_2*g*_ + 2*A*_2*u*_ + 4*E_g_* + 4*E_u_*
ν_3_, ν_4_, *F*_2_	→	3*A*	→	3*A*_1*g*_ + 3*A*_1*u*_ + 3*A*_2*g*_ + 3*A*_2*u*_ + 6*E_g_* + 6*E_u_*

**Table 4:** Electrophysical properties of isostructural ternary molybdates K_5_*R*Hf(MoO_4_)_6_ (*R* = Cr, Fe, Sc, In).

Molybdate	Conductivity, S cm^−1^ at 773 K	The Activation Energy of Electrotransfer, eV	Reference
K_5_CrHf(MoO_4_)_6_	5.22 × 10^−4^	0.73 (640–830 K)	[73]
K_5_FeHf(MoO_4_)_6_	4 × 10^−4^	0.8 (740–893 K)	this study
K_5_ScHf(MoO_4_)_6_	2.6 × 10^−4^	0.84 (750–920 K)	[31]
K_5_InHf(MoO_4_)_6_	1.59 × 10^−4^	0.85 (750–920 K)	[35]

## Data Availability

Data are available from the authors on request.

## Supplementary Materials

The following supporting information can be downloaded at: https://www.mdpi.com/article/10.3390/molecules28041629/s1, Table S1: Unit cell parameters of triple molybdates *A*_5_*MT*(MoO_4_)_6_ (*A* = K, Rb, Cs, Tl; *M* = In, Bi, *Ln*; *T* = Zr, Hf), space group R$\bar{3}$c, Z = 6; Table S2: Main parameters of processing and refinement of the powder K5FeHf(MoO_4_)_6_ sample; Table S3: Thermal properties of compounds *A*_5_*MT*(MoO_4_)_6_ (*A* = K, Rb, Cs, Tl; *M* = In, Sc, Bi, *Ln*; *T* = Zr, Hf).

## References

[1] Klevtsova R.F., Bazarova Z.G., Glinskaya L.A., Alekseev V.I., Arkhincheeva S.I., Bazarov B.G., Klevtsov P.V., Fedorov K.N. (1994). Synthesis of ternary potassium, magnesium, and zirconium molybdates. The crystal structure of K_5_(Mg_0.5_Zr_1.5_)·(MoO_4_)_6_. J. Struct. Chem..

[2] García-Cortés A., Serrano M., Zaldo C., Cascales C., Strömqvist G., Pasiskevicius V. (2008). Nonlinear refractive indices of disordered NaT(XO_4_)_2_ T=Y, La, Gd, Lu and Bi, X=Mo, W femtosecond laser crystals. Appl. Phys. B Laser Opt..

[3] Zhou D., Clive A.R., Pang L.-X., Wang H., Wu X.-G., Guo J., Zhang G.-Q., Shui L., Yao X. (2011). Microwave dielectric properties of Li_2_(M^2+^)_2_Mo_3_O_12_ and Li_3_(M^3+^)Mo_3_O_12_ (M = Zn, Ca, Al, and In) lyonsite-related-type ceramics with ultra-low sintering temperatures. J. Am. Ceram. Soc..

[4] Atuchin V., Grossman V., Adichtchev S., Surovtsev N., Gavrilova T., Bazarov B. (2012). Structural and vibrational properties of microcrystalline TlM(MoO_4_)_2_ (M = Nd, Pr) molybdates. Opt. Mater..

[5] Shi P., Xia Z., Molokeev M.S., Atuchin V.V. (2014). Crystal chemistry and luminescence properties of red-emitting CsGd_1_-xEux(MoO_4_)_2_ solid-solution phosphors. Dalton Trans..

[6] Savina A., Atuchin V., Solodovnikov S., Solodovnikova Z.A., Krylov A., Maximovskiy E., Molokeev M., Oreshonkov A., Pugachev A., Khaikina E. (2015). Synthesis, structural and spectroscopic properties of acentric triple molybdate Cs_2_NaBi(MoO_4_)_3_. J. Solid State Chem..

[7] Atuchin V., Aleksandrovsky A., Chimitova O., Diao C.-P., Gavrilova T., Kesler V., Molokeev M., Krylov A., Bazarov B., Bazarova J. (2015). Electronic structure of β-RbSm(MoO_4_)_2_ and chemical bonding in molybdates. Dalton Trans..

[8] Kurilchik S., Loiko P., Yasukevich A., Trifonov V., Volokitina A., Vilejshikova E., Kisel V., Mateos X., Baranov A., Goriev O. (2017). Orthorombic Yb:Li_2_Zn_2_(MoO_4_)_3_—A novel potential crystal for broadly tunable lasers. Laser Phys. Lett..

[9] Solodovnikov S.F., Atuchin V.V., Solodovnikova Z.A., Khyzhun O.Y., Danylenko M.I., Pishchur D.P., Plyusnin P.E., Pugachev A.M., Gavrilova T.A., Yelisseyev A.P. (2017). Synthesis, structural, thermal, and electronic properties of palmierite-related double molybdate α-Cs_2_Pb(MoO_4_)_2_. Inorg. Chem..

[10] Atuchin V.V., Aleksandrovsky A.S., Bazarov B.G., Bazarova J.G., Chimitova O.D., Denisenko Y.G., Gavrilova T.A., Krylovk A.S., Maximovskiy E.A., Molokeev M.S. (2019). Exploration of structural, vibrational and spectroscopic properties of self-activated orthorhombic double molybdate RbEu(MoO_4_)_2_ with isolated MoO4 units. J. Alloys Compd..

[11] Lim C.-S., Aleksandrovsky A., Molokeev M., Oreshonkov A., Atuchin V. (2021). Structural and spectroscopic effects of Li+ substitution for Na+ in LixNa_1_-xCaGd_0.5_Ho_0.05_Yb_0.45_(MoO_4_)_3_ scheelite-type upconversion phosphors. Molecules.

[12] Begam K., Taufiq-Yap Y., Michael M., Prabaharan S. (2004). A new NASICON-type polyanion, LixNi_2_(MoO_4_)_3_ as 3-V class positive electrode material for rechargeable lithium batteries. Solid State Ion..

[13] Chimitova O.D., Bazarov B.G., Fedorov K.N., Bazarova Z.G. (2008). Electrical properties of triple molybdates Rb_5_LnHf(MoO_4_)_6_. Russ. J. Appl. Chem..

[14] Sorokin N.I. (2009). Ionic conductivity of double sodium-scandium and cesium-zirconium molybdates. Phys. Solid State.

[15] Mikhailova D., Sarapulova A., Voss A., Thomas A., Oswald S., Gruner W., Trots D.M., Bramnik N.N., Ehrenberg H. (2010). Li_3_V(MoO_4_)_3_: A New Material for Both Li Extraction and Insertion. Chem. Mater..

[16] Souilem A., Zid M.F. (2016). Synthèse, étude et validation structurale d’un triple bis-molybdate en couches, Ag_0.60_Na_0.40_Fe(MoO_4_)_2_lié à yavapaiite. Acta Crystallogr. Sect. E Crystallogr. Commun..

[17] Kotova I.Y., Solodovnikov S.F., Solodovnikova Z.A., Belov D.A., Stefanovich S.Y., Savina A.A., Khaikina E.G. (2016). New series of triple molybdates AgA_3_R(MoO_4_)_5_ (A = Mg, R = Cr, Fe; A = Mn, R = Al, Cr, Fe, Sc, In) with framework structures and mobile silver ion sublattices. J. Solid State Chem..

[18] Mhiri M., Badri A., Ben Amara M. (2016). Synthesis and crystal structure of NaMgFe(MoO_4_)_3_. Acta Crystallogr. Sect. E Crystallogr. Commun..

[19] Sorokin N.I. (2017). Ionic conductivity of KMgCr(MoO_4_)_3_ molybdate. Crystallogr. Rep..

[20] Jendoubi I., Ptak M., Pikul A., Chmielowiec J., Ciupa A., Mączka M., Zid M.F. (2019). Synthesis, crystal structure, phonon, magnetic and electrical properties of new molybdate Na_2_Mn_2_(MoO_4_)_3_. J. Solid State Chem..

[21] Grossman V., Adichtchev S.V., Atuchin V.V., Bazarov B.G., Bazarova J.G., Kuratieva N., Oreshonkov A.S., Pervukhina N.V., Surovtsev N.V. (2020). Exploration of the structural and vibrational properties of the ternary molybdate Tl_5_BiHf(MoO_4_)_6_ with isolated MoO_4_ units and Tl+ conductivity. Inorg. Chem..

[22] Chimitova O., Bazarov B., Bazarova J., Atuchin V., Azmi R., Sarapulova A., Mikhailova D., Balachandran G., Fiedler A., Geckle U. (2021). The crystal growth and properties of novel magnetic double molybdate RbFe_5_(MoO_4_)_7_ with mixed Fe^3+^/Fe^2+^ states and 1D negative thermal expansion. CrystEngComm.

[23] BBazarov G., Klevtsova R., Bazarova T., Glinskaya L., Fedorov K., Bazarova Z. (2005). Synthesis and crystal structure of triple molybdate K_5_InHf(MoO_4_)_6_. Russ. J. Inorg. Chem..

[24] Bazarov B.G., Klevtsova R.F., Chimitova O.D., Glinskaya L.A., Fedorov K.N., Tushinova Y.L., Bazarova Z.G. (2006). Phase formation in the Rb_2_MoO_4_-Er_2_(MoO_4_)_3_-Hf(MoO_4_)_2_ system and the crystal structure of new triple molybdate Rb_3_ErHf(MoO_4_)_6_. Russ. J. Inorg. Chem..

[25] Romanova E.Y., Bazarov B.G., Klevtsova R.F., Glinskaya L.A., Tushinova Y.L., Fedorov K.N., Bazarova Z.G. (2007). Phase formation in the K_2_MoO_4_-Lu_2_(MoO_4_)-Hf(MoO_4_)_2_ system and the structural study of triple molybdate K_5_LuHf(MoO_4_)_6_. Russ. J. Inorg. Chem..

[26] Chimitova O.D., Bazarov B.G., Klevtsova R.F., Fedorov K.N., Glinskaya L.A., Kuznetsov M.V., Bazarova Z.G. (2007). Synthesis, crystal structure, and electrical properties of the new ternary molybdate Rb_5_NdHf(MoO_4_)_6_. Russ. Chem. Bull..

[27] Bazarov B.G., Namsaraeva T.V., Klevtsova R.F., Anshits A., Vereshchagina T.A., Kurbatov R.V., Glinskaya L.A., Fedorov K.N., Bazarova Z.G. (2008). Phase equilibrium in the Cs_2_MoO_4_-Bi_2_(MoO_4_)_3_-Zn(MoO_4_)_2_ system and the crystal structure of new triple molybdate Cs_5_BiZr(MoO_4_)_6_. Russ. J. Inorg. Chem..

[28] Bazarov B.G., Chimitova O.D., Klevtsova R.F., Tushinova Y.L., Glinskaya L.A., Bazarova Z.G. (2008). Crystal structure of a new ternary molybdate in the Rb_2_MoO_4_-Eu_2_(MoO_4_)_3_-Hf(MoO_4_)_2_ system. J. Struct. Chem..

[29] Chimitova O., Bazarov B., Klevtsova R., Anshits A., Fedorov K., Dubentsov A., Vereshchagina T., Tushinova Y., Glinskaya L., Bazarova Z. (2010). Crystal structure of triple molybdate in the Rb_2_MoO_4_–Nd_2_(MoO_4_)_3_–Zr(MoO_4_)_2_ system. J. Struct. Chem..

[30] Gongorova L., Bazarov B., Chimitova O., Anshits A., Vereschagina T., Klevtsova R., Glinskaya L., Bazarova Z. (2012). Crystal structure of a new ternary molybdate Rb_5_CeZr(MoO_4_)_6_. J. Struct. Chem..

[31] Grossman V.G., Bazarova J.G., Molokeev M.S., Bazarov B.G. (2020). New triple molybdate K5ScHf(MoO_4_)_6_: Synthesis, properties, structure and phase equilibria in the M_2_MoO_4_–Sc_2_(MoO_4_)_3_–Hf(MoO_4_)_2_ (M = Li, K) systems. J. Solid State Chem..

[32] Grossman V.G., Bazarov B.G., Bazarova Z.G. (2008). Subsolidus phase diagrams for the Tl_2_MoO_4_-Ln_2_(MoO_4_)_3_-Hf(MoO_4_)_2_ systems, where Ln = La-Lu. Russ. J. Inorg. Chem..

[33] Logvinova A., Bazarov B., Tushinova Y., Bazarova J. (2017). Phase Relations in the K_2_MoO_4_–Ln_2_(MoO_4_)_3_–Zr(MoO_4_)_2_ (Ln = La–Lu, Y) systems. Inorg. Mater..

[34] Grossman V., Bazarov B., Bazarova T., Glinskay L., Bazarova J., Temuujin J. (2017). Phase equilibria in the Tl_2_MoO_4_–Ho_2_(MoO_4_)_3_–Zr(MoO_4_)_2_ system and the crystal structure of Ho_2_Zr_2_(MoO_4_)_7_ and TlHoZr_0.5_(MoO_4_)_3_. J. Ceram. Proc. Res..

[35] Grossman V., Bazarov B., Bazarova J. (2019). K_5_InHf(MoO_4_)_6_: A solid state conductor. IOP Conf. Ser. Earth Environ. Sci..

[36] Shannon R.D. (1976). Revised effective ionic radii and systematic studies of interatomic distances in halides and chalcogenides. Acta Cryst. A.

[37] Andersson A., Kalska B., Eyob P., Aernout D., Häggström L., Thomas J. (2001). Lithium insertion into rhombohedral Li_3_Fe_2_(PO_4_)_3_. Solid State Ion..

[38] Kotova I.Y., Kozhevnikova N.M. (2003). Phase Relations and Electrical Properties of Phases in Systems Na_2_MoO_4_-AMoO_4_-R_2_(MoO_4_)_3_(A = Mg, Mn, Co, Ni; R = Cr, Fe). Russ. J. Appl. Chem..

[39] Grossman V.G., Bazarov B.G., Klevtsova R.F., Glinskaya L.A., Bazarova Z.G. (2012). Phase equilibria in the Tl_2_MoO_4_-Fe_2_(MoO_4_)_3_-Hf(MoO_4_)_2_ system and the crystal structure of ternary molybdate Tl(FeHf_0.5_)(MoO_4_)_3_. Russ. Chem. Bull..

[40] Atuchin V., Vinnik D., Gavrilova T., Gudkova S., Isaenko L., Jiang X., Pokrovsky L., Prosvirin I., Mashkovtseva L., Lin Z. (2016). Flux crystal growth and the electronic structure of BaFe_12_O_19_ hexaferrite. J. Phys. Chem. C.

[41] Vinnik D., Prosvirin I., Zhivulin V., Wang N., Jiang X., Trofimov E., Zaitseva O., Gudkova S., Nemrava S., Zherebtsov D. (2020). Crystal growth, structural characteristics and electronic structure of Ba_1_-xPbxFe_12_O_19_ (x = 0.23–0.80) hexaferrites. J. Alloys Compd..

[42] Ramana C.V., Bandi M., Nair A., Manciu F.S., Sreenivasan S., Shutthanandan V. (2021). Electronic structure, chemical bonding, and electrocatalytic activity of Ba(Fe_0.7_Ta_0.3_)O_3_−δ compounds. ACS Appl. Energy Mater..

[43] Pinaeva L., Prosvirin I., Chesalov Y., Atuchin V. (2022). High-temperature abatement of N_2_O over FeOx/CeO_2_-Al_2_O_3_ catalysts: The effects of oxygen mobility. Catalysts.

[44] Trunov K., Efremov V., Velikodny Y. (1986). Crystal Chemistry and Properties of Double Molybdates and Tungstates.

[45] Mokhosoev M., Alekseev F., Lutsyk V. (1978). State Diagrams of Molybdate and Tungstate Systems.

[46] Klevtsova R., Klevtsov P. (1970). Synthesis and crystal structure of the double moibdates KR(MoO_4_)_2_ for R_3_+ = Al, Sc, and Fe, and of the tungstate KSc(WO_4_)_2_. Sov. Phys. Crystallogr. (Engl. Transl.).

[47] Kadyrova Y. (2010). Abstract of the Dissertation… Candidate of Chemical Sciences.

[48] Lazoryak B., Efremov V. (1987). The double molybdates Me_5_Tr(MoO_4_)_4_. Kristallografiya.

[49] Tissot R.G., Rodriguez M.A., Sipola D.L., Voigt J.A. (2001). X-ray powder diffraction study of synthetic Palmierite, K_2_Pb(SO_4_)_2_. Powder Diffr..

[50] Otko A.I., Nesterenko N.M., Povstyanyi L.V. (1978). Phenomenological approach to structural phase transitions in trigonal double molybdates and tungstates. Phys. Status Solidi (A).

[51] Nesterenko N.M., Fomin V.I. (1979). Soft modes and the raman spectrum in KSc(MoO_4_)_2_ at ferroelastic phase transitions. Phys. Status Solidi (A).

[52] Zapart W., Zapart M.B. (1990). Incommensurate phase in KSc(MoO_4_)_2_ by EPR of Cr^3+^. Phys. Status Solidi (A).

[53] Zapart W. (1990). Possibility of simultaneously incommensurate and ferroelastic phase in RbIn(MoO_4_)_2_ by EPR of Cr^3+^ ion. Phys. Status Solidi (A).

[54] Zapart M.B., Zapart W. (1993). Investigations of incommensurate phase in KSc(WO_4_)_2_. Phase Transit..

[55] Svistov L.E., Smirnov A.I., Prozorova L.A., Petrenko O.A., Shapiro A.Y., Dem’Yanets L.N. (2004). On the possible coexistence of spiral and collinear structures in antiferromagnetic KFe(MoO_4_)_2_. J. Exp. Theor. Phys. Lett..

[56] Maczka M., Pietraszko A., Saraiva G.D., Filho A.G.S., Paraguassu W., Lemos V., A Perottoni C., Gallas M.R., Freire P.T.C., E Tomaszewski P. (2005). High pressure effects on the structural and vibrational properties of antiferromagnetic KFe(MoO_4_)_2_. J. Phys. Condens. Matter.

[57] Maczka M., Pietraszko A., Paraguassu W., Filho A.G.S., Freire P.T.C., Filho J.M., Hanuza J. (2009). Structural and vibrational properties of K_3_Fe(MoO_4_)_2_(Mo_2_O_7_)—A novel layered molybdate. J. Phys. Condens. Matter.

[58] Zolotova E. (1986). Abstract of the Dissertation… Candidate of Chemical Sciences.

[59] Tushinova Y., Bazarova J., Arhincheeva S. (2002). Phase equilibria in R_2_(MoO_4_)_3_–Zr(MoO_4_)_2_ systems. All-Russian Scientific Conference with International Participation.

[60] Bruker AXS (2008). TOPAS V4: General Profile and Structure Analysis Software for Powder Diffraction Data—User’s Manual.

[61] Gongorova L. (2012). Phase Equilibrium, Structure and Properties of New Molybdates in the Systems Rb_2_MoO_4_–Ln_2_(MoO_4_)_3_–Zr(MoO_4_)_2_ (Ln = La–Lu). Ph.D. Thesis.

[62] Lim C.S., Aleksandrovsky A., Atuchin V., Molokeev M., Oreshonkov A. (2020). Microwave-Employed Sol–Gel Synthesis of Scheelite-Type Microcrystalline AgGd(MoO_4_)_2_:Yb^3+^/Ho^3+^ Upconversion Yellow Phosphors and Their Spectroscopic Properties. Crystals.

[63] Tillard M., Granier D., Daenens L., Reibel C., Armand P. (2022). Crystal structure, Raman characterization, and magnetic properties of the hydrate RbYb(MoO_4_)_2_, H_2_O. J. Solid State Chem..

[64] Armand P., Granier D., Reibel C., Tillard M. (2022). Growth, crystal structure, and properties of the Li_3_Ba_2_Ln_3_(MoO_4_)_8_ (Ln = Er, Tm) molybdates. Solid State Sci..

[65] Reshak A.H., Alahmed Z.A., Bila J., Atuchin V.V., Bazarov B.G., Chimitova O.D., Molokeev M.S., Prosvirin I.P., Yelisseyev A.P. (2016). Exploration of the Electronic Structure of Monoclinic α-Eu_2_(MoO_4_)_3_: DFT-Based Study and X-ray Photoelectron Spectroscopy. J. Phys. Chem. C.

[66] Malakhovskii A.V., Sukhachev A.L., Vasil’Ev A.D., Leont’Ev A.A., Kartashev A.V., Temerov V.L., Gudim I.A. (2012). Nature of optical properties of GdFe_3_(BO_3_)_4_ and GdFe_2.1_Ga_0.9_(BO_3_)_4_ crystals and other 3d5 antiferromagnets. Eur. Phys. J. B.

[67] Voronkova V., Kharitonova E., Orlova E., Gorshkov N., Goffman V. (2017). Synthesis and Unusual Properties of Tetragonal Pb-Contained Oxymolybdates Based on La_2_MoO_6_. Eur. J. Inorg. Chem..

[68] Zouaoui M., Jendoubi I., Zid M.F., Bourguiba N.F. (2021). Synthesis, crystal structure and physico-chemical investigations of a new lyonsite molybdate Na_0.24_Ti_1.44_(MoO_4_)_3_. J. Solid State Chem..

[69] Fedorov D., Buzlukov A., Baklanova Y., Suetin D., Tyutyunnik A., Korona D., Maksimova L., Ogloblichev V., Denisova T., Medvedeva N. (2022). Sodium diffusion in scheelite-type Na_2_Zr(MoO_4_)_3_ and Na_4_Zr(MoO_4_)_4_. Ceram. Int..

[70] Ben Nasr W., Mahmoud A., Boschini F., Ben Rhaiem A. (2019). Optical and AC conductivity studies on Li_2_-xRbx MoO_4_ (x = 0, 0.5, 1) compounds. J. Alloys Compd..

[71] Nasri R., Larbi T., Amlouk M., Zid M.F. (2019). Highly efficient K_0.4_Na_3.6_Co(MoO_4_)_3_ new alluaudite type structure for photocatalytic degradation of methylene blue and green diamine B dyes. J. Mater. Sci. Mater. Electron..

[72] Grossman V.G., Bazarova J.G., Molokeev M.S., Bazarov B.G. (2020). Thallium ionic conductivity of new thallium indium hafnium molybdate ceramics. Ionics.

[73] Grossman V.G., Molokeev M.S., Bazarov B.G., Bazarova J.G. (2021). Potassium and thallium conductors with a trigonal structure in the M_2_MoO_4_–Cr_2_(MoO_4_)_3_–Hf(MoO_4_)_2_ (M = K, Tl) systems: Synthesis, structure, and ionic conductivity. J. Alloys Compd..

[74] Grossman V.G., Molokeev M.S., Bazarova J.G., Bazarov B.G., Sorokin N.I. (2021). Structural, thermal and electrical studies of thallium-scandium-hafnium(zirconium) molybdates. J. Solid State Chem..

[75] Namsaraeva T., Bazarov B., Mikhailova D., Kuratieva N., Sarapulova A., Senyshyn A., Ehrenberg H. (2011). Orthomolybdates in the Cs-FeII,III-Mo-O system: Cs_4_Fe(MoO_4_)_3_, Cs_2_Fe_2_(MoO_4_)_3_ and CsFe_5_(MoO_4_)_7_. Eur. J. Inorg. Chem..

[76] Chen H.-Y. (1979). The crystal structure and twinning behavior of ferric molybdate, Fe_2_(MoO_4_)_3_. Mater. Res. Bull..

[77] Auray M., Quarton M., Tarte P. (1987). Crystal data for two molybdates M(MoO_4_)_2_ with M = Zr, Hf. Power Diffr..

[78] Bruker AXS Inc. (2004). APEX2 (Version 1.08), SAINT (Version 7.03), and SADABS (Version 2.11). Bruker Advanced X-ray Solutions.

[79] Sheldrick G.M. (2015). Crystal structure refinement with SHELXL. Acta Crystallogr. Sect. C Struct. Chem..

[80] Clark S.J., Segall M.D., Pickard C.J., Hasnip P.J., Probert M.I.J., Refson K., Payne M.C. (2005). First Principles Methods Using CASTEP. Z. Kristallogr. Cryst. Mater..

[81] Perdew J.P., Ruzsinszky A., Csonka G.I., Vydrov O.A., Scuseria G.E., Constantin L.A., Zhou X., Burke K. (2008). Restoring the Density-Gradient Expansion for Exchange in Solids and Surfaces. Phys. Rev. Lett..

[82] Monkhorst H.J., Pack J.D. (1976). Special points for Brillouin-zone integrations. Phys. Rev. B.

