# Supplementary material for: Structural, Spectroscopic, Electric and Magnetic Properties of New Trigonal K5FeHf(MoO4)6 Orthomolybdate
Source: Molecules. 2023 Feb 8;28(4):1629. doi: 10.3390/molecules28041629 (PMC9961824; doi:10.3390/molecules28041629)
Supplement: Supplementary file 1 [file molecules-28-01629-s001.zip › molecules-2157225-supplementary.pdf]

Supplementary Information

Structural, Spectroscopic, Electric and Magnetic Properties of New Trigonal K5FeHf(MoO4)6 Orthomolybdate

Victoria Grossman [1], Victor Atuchin [2,3,4,5,6,*], Bair G. Bazarov [1], Aleksandr Aleksandrovsky [7,8], Evgeniy Eremin [9,10], Alexander Krylov [11], Natalia Kuratieva [12], Jibzema G. Bazarova [1], Nikolai Maximov [13], Maxim Molokeev [9,10,14], Aleksandr Oreshonkov [11,15], Natalia Pervukhina [12] and Nikolay Shestakov [11]

1 Laboratory of Oxide Systems, Baikal Institute of Nature Management, SB RAS, Ulan-Ude 670047, Russia
2 Laboratory of Optical Materials and Structures, Institute of Semiconductor Physics, SB RAS, Novosibirsk 630090, Russia
3 Department of Applied Physics, Novosibirsk State University, Novosibirsk 630090, Russia
4 Research and Development Department, Kemerovo State University, Kemerovo 650000, Russia
5 Department of Industrial Machinery Design, Novosibirsk State Technical University, Novosibirsk 630073, Russia
6 R&D Center "Advanced Electronic Technologies", Tomsk State University, Tomsk 634034, Russia
7 Laboratory of Coherent Optics, Kirensky Institute of Physics, Federal Research Center KSC SB RAS, Krasnoyarsk 660036, Russia
8 Institute of Nanotechnology, Spectroscopy and Quantum Chemistry, Siberian Federal University, Krasnoyarsk 660041, Russia
9 Kirensky Institute of Physics, Federal Research Center KSC SB RAS, Krasnoyarsk 660036, Russia
10 School of Engineering Physics and Radio Electronics, Siberian Federal University, Krasnoyarsk 660041, Russia
11 Laboratory of Molecular Spectroscopy, Kirensky Institute of Physics, Federal Research Center KSC SB RAS, Krasnoyarsk 660036, Russia
12 Laboratory of Crystal Chemistry, Institute of Inorganic Chemistry, SB RAS, Novosibirsk 630090, Russia
13 Institute of Chemistry and Chemical Technology, Federal Research Center KSC SB RAS, Krasnoyarsk 660036, Russia
14 Department of Physics, Far Eastern State Transport University, Khabarovsk 680021, Russia
15 School of Engineering and Construction, Siberian Federal University, Krasnoyarsk 660041, Russia
* Correspondence: atuchin@isp.nsc.ru; Tel.: +7-(383)-330-8889

Table S1. Unit cell parameters of triple molybdates $A_5MT(MoO_4)_6$ (A = K, Rb, Cs, Tl; M = In, Bi, Ln; T = Zr, Hf), space group $R\bar{3}c$, Z =6

Compound	$a = b$, Å	c, Å	V, Å^3	M1	M2	Reference
$Cs_5BiZr(MoO_4)_6$	10.9569(2)	39.804(4)	4138.4(4)	0.919Bi+0.081Zr	0.919Zr+0.081Bi	27
$Rb_5NdHf(MoO_4)_6$	10.7550(2)	38.8427(13)	3891.0(2)	0.904Nd+0.096Hf	0.904Hf+0.096Nd	26
$Rb_{4.7}Nd_{0.7}Zr_{1.3}(MoO_4)_6$	10.7561(2)	38.7790(12)	3885.41(16)	0.6Nd+0.4Zr	0.1Nd+0.9Zr	29
$Rb_5ErHf(MoO_4)_6$	10.7511(1)	38.6543(7)	3869.31(9)	1Er	1Hf	24
$Rb_5CeZr(MoO_4)_6$	10.7248(2)	38.796(1)	3864.52(14)	0.834Ce+0.166Zr	0.834Zr+0.166Ce	30
$Rb_{4.98}Eu_{0.86}Hf_{1.11}(MoO_4)_6$	10.7264(1)	38.6130(8)	3847.44(9)	0.86Er+0.11Hf	1Hf	28
$Tl_5BiHf(MoO_4)_6$	10.6801(4)	38.5518(14)	3808.3(2)	0.92Bi+0.08Hf	0.92Hf +0.08Bi	21
$K_5ScHf(MoO_4)_6$	10.56312(8)	37.6251(3)	3635.74(6)	0.548Hf+0.452Sc	0.548Sc+0.452Hf	31
$K_5LuHf(MoO_4)_6$	10.6536(1)	37.8434(8)	3719.75(9)	0.65Lu+0.35Hf	0.65Hf+0.35Lu	25
$K_5InHf(MoO_4)_6$	10.564(1)	37.632(4)	3637.0(6)	0.413Hf+0.587In	0.413In+0.587Hf	23
$K_5FeHf(MoO_4)_6$	10.4633(3)	37.3113(9)	3537.60(17)	0.732Hf+0.268Fe	0.732Fe+0.268Hf	This study

Table S2. Main parameters of processing and refinement of the powder $K_5FeHf(MoO_4)_6$ sample

Compound	$K_5FeHf(MoO_4)_6$
Sp. Gr.	$R\bar{3}c$
a (Å)	10.4837(3)
c (Å)	37.3492(11)
V (Å^3)	3555.0(2)
Z	6
2θ-interval, º	8-100
R_{wp} ,%	9.87
R_p ,%	7.76
χ^2	1.09
R_B ,%	3.33

Table S3. Thermal properties of compounds $A_5MT(MoO_4)_6$ (*A* = K, Rb, Cs, Tl; *M* = In, Sc, Bi, *Ln*; *T* = Zr, Hf)

Compound	T_{pt}, K	T_m, K	ΔH_{pt}, J/g	ΔH_m, J/g	Reference
$K_5DyZr(MoO_4)_6$	778	893	-	-	33
$K_5HoZr(MoO_4)_6$	777	913	-	-	33
$K_5YZr(MoO_4)_6$	769	927	-	-	33
$K_5ErZr(MoO_4)_6$	770	929	-	-	33
$K_5TmZr(MoO_4)_6$	753	952	-	-	33
$K_5YbZr(MoO_4)_6$	752	969	-	-	33
$K_5LuZr(MoO_4)_6$	752	981	-	-	33
$K_5InHf(MoO_4)_6$	910	1015	−1.279	−44.48	35
$K_5ScHf(MoO_4)_6$	-	999	-	-	31
$K_5FeHf(MoO_4)_6$	835	957	−0.45	−101	This study
$Rb_5CeZr(MoO_4)_6$	760, 809	883	-	-	68
$Rb_5NdZr(MoO_4)_6$	742, 813	928	-	-	68
$Rb_5TbZr(MoO_4)_6$	735, 817	936	-	-	68
$Rb_5ErZr(MoO_4)_6$	821	952	-	-	68
$Tl_5HoZr(MoO_4)_6$	721,819	852	-	-	34
$Tl_5BiHf(MoO_4)_6$	731	871	−3.15	−41.71	21
